# Insect fat influences broiler performance, meat quality, and the cecal microbiota similarly to plant oils rather than animal fats

**DOI:** 10.1038/s41598-025-02820-3

**Published:** 2025-05-24

**Authors:** Muhammad Rumman Aslam, Bartosz Kierończyk, Mateusz Rawski, Piotr Szymkowiak, Kinga Stuper-Szablewska, Paweł Kołodziejski, Robert Mikuła, Agata Dankowiakowska, Damian Józefiak

**Affiliations:** 1 Department of Animal Nutrition, Faculty of Veterinary Medicine and Animal Science, Poznań University of Life Sciences, Wołyńska 33, Poznań, 60-637, Poland; 2 Department of Zoology, Laboratory of Inland Fisheries and Aquaculture, Faculty of Veterinary Medicine and Animal Science, Poznań University of Life Sciences, Wojska Polskiego 71c, Poznań, 60-625, Poland; 3 Department of Chemistry, Faculty of Wood Technology, Poznań University of Life Sciences, Wojska Polskiego 75, Poznań, 60-625, Poland; 4 Department of Animal Physiology, Biochemistry, and Biostructure, Faculty of Veterinary Medicine and Animal Science, Poznań University of Life Sciences, Wołyńska 35, Poznań, 60-637 Poland; 5 Department of Animal Physiology and Physiotherapy, Faculty of Animal Breeding and Biology, Bydgoszcz University of Science and Technology, Mazowiecka 28, Bydgoszcz, 85-084 Poland

**Keywords:** Chicken, Dietary fats, Black soldier fly, Physiological response, Immune response, Consumer acceptance, Animal physiology, Fat metabolism, Feeding behaviour, Enzymes, Lipids, Adaptive immunity, Antimicrobial responses, Bacteria, Metabolism, Gastrointestinal system, Large intestine, Liver, Microbiota, Small intestine, Stomach

## Abstract

We evaluated the addition of *Hermetia illucens* larvae (BSFL) fat to broiler diets compared to that of soybean oil (SO), rapeseed oil (RO), palm oil (PO), palm kernel fatty acid distillate (PKFD), poultry fat (PF), pig lard (PL), and beef tallow (BT) on performance, meat quality, and cecal microbiota abundance. BSFL addition reduced the feed conversion ratio compared to PO, PF, and BT addition and improved nutrient digestibility at 14 days and 28 days of age. BSFL addition enhanced broiler immune responses compared with SO, PO, and PL by increasing IgA levels; compared to PO by increasing IgG levels; and compared to PL by increasing IgY and IL-6 levels. Moreover, broiler free triiodothyronine levels increased when BSFL was added. Reduced drip loss after 7 days was observed in the RO, PO, PF, PL, and BT groups, while the salt-induced water uptake in the PL group was greater than that in the BSFL group. Compared with the SO group, BSFL addition increased C18:3 and overall n-3 FAs in breast meat. BSFL fat in broiler diets leads to outcomes that are more similar to those of plant oils than to those of animal fats, particularly regarding nutrient digestiblity and immune response.

**Supplementary Information:**

The online version contains supplementary material available at 10.1038/s41598-025-02820-3.

## Introduction

Insects are increasingly being recognized as sustainable feed alternatives for animal nutrition because of their nutrient content and relatively low environmental impact^1^. Their biomass is rich in crude protein (30–70%) and crude fat (10–50%)^2^, rendering them viable substitutes for conventional feed materials^3^. Specifically, the dietary fat derived from black soldier fly (*Hermetia illucens*) larvae (BSFL) is favored because of its substantial metabolizable energy level^4^ and noteworthy concentration of medium-chain fatty acids (MCFAs), specifically lauric acid (C12:0)^3^. MCFAs are known for their simple digestibility and efficient absorption^5^, and they are directly absorbed through simple diffusion by intestinal enterocytes^6^ independent of bile secretion^7^. Moreover, MCFAs, especially lauric acid, have antimicrobial properties and support overall gut health^1,6^.

Dietary fats sourced from plants and animals significantly affect broiler performance, including immune function, meat quality, and the gut microbiota^8,9^. Additionally, numerous studies have investigated the effects of various levels of BSFL dietary fats on poultry, collectively revealing that its incorporation into broiler diets has substantial potential for enhancing growth performance, nutrient digestibility, meat quality, and gut microbiota composition without any detrimental impact on gut histology^10–13^. Furthermore, the implementation of BSFL fats significantly impacts the fatty acid (FA) profile of breast muscle, including a pronounced effect on n-3 FA deficiency^14^.

There is a limited understanding regarding how fats from BSFL compare with other common dietary fats in broiler nutrition. Therefore, the present study focused on the direct comparison of BSFL fat as a reference group to eight selected dietary fats in broiler nutrition. In the available literature, the differences between dietary fats (of plant and animal origin) have frequently been studied; however, BSFLs have not been compared with a wide spectrum of energy source ingredients. On the basis of our knowledge, the current study is the first to evaluate the effects of BSFL dietary fat inclusion in broiler chicken diets, emphasizing comparisons with a broad range of commonly used plant oils and animal fats in terms of growth performance, nutrient utilization, internal organ morphometry, liver histomorphology, selected serum biochemical and immunological indices, hormone concentrations, cecal microbiota populations, carcass characteristics, breast muscle and liver quality, and sensory meat analyses.

## Results

### Growth performance

In general, no significant (*P* > 0.05) change in body weight gain (BWG) was observed throughout the entire experiment (Table 1), except in the first week, when feeding PKFD increased (*P* = 0.006) the BWG compared with that in the broilers fed BSFL fat. For the feed intake (FI), no differences (*P* > 0.05) were found between animals fed BSFL and those fed other selected dietary fat sources. The feed conversion ratios (FCRs) displayed significant (*P* < 0.05) variations during the 1st (1–7 days), 3rd (15–21 days), and 5th (28–35 days) weeks, and throughout the entire period (1–35 days). During the first week, the FCR was lower (*P* = 0.016) in the PKFD treatment compared to the BSFL group. By the third week, the FCR increased (*P* < 0.001) in the PF and BT groups. Similarly, in the fifth week, the FCR increased (*P* < 0.001) in the RO, PO, and PF groups, whereas no significant changes (*P* > 0.05) were observed in the other treatments compared with BSFL during these periods. Over the entire 35-day period, broilers fed PO, PF, or BT presented a significant increase in FCR (*P* < 0.001). In contrast, FCR in the SO, RO, PKFD, and PL groups remained comparable (*P* > 0.05) to that of BSFL treatment.

**Table 1:** Effects of various dietary fat applications in broiler chicken diets on growth performance.

	Treatment								^9^SEM	*P* value
	^1^BSFL	^2^SO	^3^RO	^4^PO	^5^PKFD	^6^PF	^7^PL	^8^BT		
^10^BWG, g										
1–7 days	163	161	163	163	174*	161	159	164	0.98	0.006
8– 14 days	256	263	255	251	256	256	253	251	1.10	0.053
15–21 days	562	562	560	566	544	569	559	557	1.89	0.565
22–28 days	600	627	576	603	640	626	608	587	4.60	> 0.050
28–35 days	638	652	614	617	640	635	630	624	4.17	0.159
1–35 days	2219	2265	2168	2200	2254	2246	2210	2182	8.65	0.441
^11^FI, g										
1–7 ddays	175	174	174	175	178	173	173	175	0.65	0.830
8–14 days	333	336	332	334	332	338	333	334	1.06	0.689
15–21 days	752	753	750	768	732	775	754	769	2.91	0.144
22–28 days	901	921	885	916	934	938	911	906	4.58	0.317
28–35 ddays	977	1015	982	993	989	1016	1000	988	5.07	0.599
1–35 days	3137	3199	3122	3187	3165	3241	3171	3172	10.6	0.269
^12^FCR, g:g										
1–7 days	1.08	1.08	1.07	1.07	1.03*	1.08	1.09	1.07	0.01	0.016
8–14 days	1.30	1.28	1.30	1.33	1.30	1.32	1.32	1.33	0.01	0.004
15–21 days	1.34	1.34	1.34	1.36	1.35	1.36*	1.35	1.38***	< 0.01	< 0.001
22–28 ddays	1.50	1.47	1.54	1.52	1.46	1.50	1.50	1.55	0.01	0.001
28–35 days	1.53	1.56	1.60*	1.61**	1.54	1.60**	1.59	1.58	0.01	< 0.001
1–35 days	1.41	1.41	1.44	1.45*	1.40	1.44*	1.44	1.45***	< 0.01	< 0.001

*p < 0.05, **p < 0.01, ***p < 0.001—the reference group (BSFL) is significantly different within a row when asterisks are present. ^1^BSFL—basal diet with 100% black soldier fly (*H. illucens*) larval fat; ^2^SO—basal diet with 100% soybean oil; ^3^RO—basal diet with 100% rapeseed oil; ^4^PO—basal diet with 100% palm oil; ^5^PKFD—basal diet with 100% palm kernel fat distillate; ^6^PF—basal diet with 100% poultry fat; ^7^PL—basal diet with 100% pig lard; ^8^BT—basal diet with 100% beef tallow; ^9^SEM—standard error of the mean; ^10^BWG—body weight gain; ^11^FI—feed intake; ^12^FCR—feed conversion ratio.

The means represent 10 pens of 15 birds each until 14 days and 10 birds from 15 days (n = 10).

Analysis of variance (ANOVA) or the Kruskal‒Wallis test, depending on the normal distribution of the data, provides the probability level (*P value*) presented in the last column.

### Nutrient retention and digestibility

Calculations performed on the 14th day indicated an increase (*P* < 0.001) in dry matter (DM) digestibility in broiler fed SO, which decreased with BT feeding (Table 2). However, nitrogen retention decreased (*P* < 0.001) in the BT group, and the apparent metabolizable energy corrected to zero nitrogen balance (AME_N_) decreased (*P* < 0.001) for both the PL and BT groups compared with the BSFL group. Additionally, on the 28th day, differences were observed for the ether extract (EE) (*P* < 0.001), which was decreased in the SO group, and the AME_N_ value (*P* = 0.008) was reduced by the PL and BT treatments, in contrast to that of the BSFL group. By the end of the experiment (35th day), there was no notable change (*P* > 0.05) in the coefficient of ileal digestibility (CAID) values of DM, EE, CP, and apparent ileal digestible energy (AIDE). Furthermore, no differences (*P* > 0.05) were detected in the total tract amino acid digestibility coefficient (35 day) between animals fed BSFL and those fed other selected dietary fat sources (Supplementary Table S1). The activity of each selected pancreatic enzyme (trypsin, lipase, or amylase) did not change (*P* > 0.05) between the reference group and the experimental treatment groups.

**Table 2:** Effects of the application of various dietary fat sources to broiler chicken diets on total tract nitrogen retention, digestibility of ether extract or the crude protein coefficient, apparent ileal digestible energy (AIDE), apparent metabolizable energy corrected to zero nitrogen balance (AME_N_) and selected pancreatic enzyme activities (35d).

	Treatment								^9^SEM	*P* value
	^1^BSFL	^2^SO	^3^RO	^4^PO	^5^PKFD	^6^PF	^7^PL	^8^BT		
14 days										
Dry matter, %	75.29	77.98*	76.60	77.14	77.59	76.88	73.08	69.45***	0.48	< 0.001
Ether extract, %	95.82	95.56	93.01	93.68	96.82	93.59	95.03	92.47	0.30	0.053
N retention, %	67.39	70.67	68.68	69.84	69.83	70.30	66.44	60.56***	0.59	< 0.001
AME_N_, kcal	3310	3315	3267	3294	3270	3301	3179**	2893***	22.8	< 0.001
28 days										
Dry matter, %	78.29	79.78	79.26	78.89	83.43	81.02	75.04	75.71	0.51	0.086
Ether extract, %	89.83	81.41**	78.23	81.70	97.39	85.22	86.69	81.97	1.16	< 0.001
N retention, %	70.34	73.07	72.60	72.79	76.31	75.66	67.11	69.92	0.61	0.380
AME_N_, kcal	3379	3306	3307	3328	3473	3418	320*	3078***	22.5	0.008
35 days										
Dry matter, %	78.93	76.64	76.91	76.56	78.32	78.46	75.81	78.25	0.42	0.874
Ether extract, %	95.85	93.50	91.58	91.59	96.81	92.93	92.62	93.68	0.53	0.099
Crude protein, %	80.46	79.59	78.53	79.22	82.28	81.25	79.29	81.52	0.55	0.405
AIDE, kcal	3403	3203	3204	3230	3247	3326	3215	3184	20.7	0.156
The pancreatic enzymes activities										
Trypsin, %	100	97.2	105	109	104	89.9	89.6	110	2.89	0.500
Lipase, %	100	91.7	90.5	89.5	91.5	90.0	91.0	97.4	3.08	0.989
Amylase, %	100	104	103	97.9	98.6	93.6	90.4	104	3.51	0.980

*p < 0.05, **p < 0.01, ***p < 0.001—the reference group (BSFL) is significantly different within a row when asterisks are present.

^1^BSFL—basal diet with 100% black soldier fly (*H. illucens*) larval fat; ^2^SO—basal diet with 100% soybean oil; ^3^RO—basal diet with 100% rapeseed oil; ^4^PO—basal diet with 100% palm oil; ^5^PKFD—basal diet with 100% palm kernel fat distillate; ^6^PF—basal diet with 100% poultry fat; ^7^PL—basal diet with 100% pig lard; ^8^BT—basal diet with 100% beef tallow; ^9^SEM—standard error of the mean.

The means represent the 10 birds’ excreta/digesta pooled by 2 (*n* = 5) for total tract nitrogen retention, digestibility of EE and CP coefficient, AIDE, and AME_N_, or 8 birds’ duodenal digesta (*n* = 8) for selected pancreatic enzyme activities. Analysis of variance (ANOVA) or the Kruskal‒Wallis test, depending on the normal distribution of the data, provides the probability level (*P* value) presented in the last column.

### Selected organ weights and lengths

The selected organ weights, expressed as percentages of body weight (% of BW), along with the immune organ indexes (% of BW), and selected gastrointestinal tract (GIT) segment lengths (cm kg^−1^ BW) were calculated (Supplementary Table S2). The only differences (*P* < 0.001) were observed in the jejunum, where PKFD feeding decreased the jejunal weight compared with that following BSFL feeding.

### Liver histomorphology

**Fig. 1:**
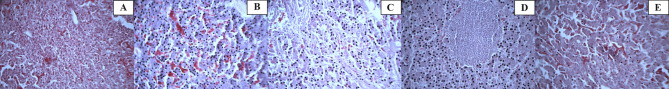
Light micrographs of broiler chicken liver tissues (hematoxylin and eosin stain, H&E). (**a**) presence of vacuoles (40×); (**b**) presence of congestions (40×); (**c**) presence of fibrosis (40×); (**d**) presence of necrosis (40 ×); (**e**) presence of parenchymal eclipse (40×).

No differences were detected in parenchymal eclipse (*P* = 0.201), the presence of vacuoles (*P* = 0.157), the number of congestions (*P* = 0.506), the number of necrotic lesions (*P* = 0.012 ANOVA, *P* > 0.05 post hoc test), or the number of fibrotic lesions (*P* = 0.146) between animals fed BSFL and those fed other dietary fat sources (Supplementary Table S3). Fat vacuolization was not detected in any treatment group; thus, the table does not show this parameter. Figure 1 provides visual examples of the analyzed samples.

### Selected serum biochemical and physiological indices

The administration of dietary fats in broiler diets had effects on selected serum biochemical parameters in the animals, with significant differences detected for aspartate aminotransferase (AST) (*P* = 0.022) and alkaline phosphatase (ALP) (*P* = 0.001). The AST concentration was reduced by PF group, whereas the ALP level was lower in the PO and PKFD treatment than in the BSFL group (Table 3). Additionally, differences were observed for all the selected immune indices except TNF-α (*P* = 0.426). A decrease in IgA (*P* = 0.022) in the SO, PO, and PL groups was noted; however, a reduced level of IgG (P = 0.007) in the PO group and lower values of IgY (*P* = 0.002) and IL-6 (*P* = 0.008) in the PL group were observed in contrast to those in the BSFL treatment. However, regarding hormone levels, a difference was noted only for free triiodothyronine (FT3) (*P* < 0.001) in the BSFL group (Table 3). Additionally, in contrast to those in the BSFL group, the FT3 concentrations of animals fed each of the tested fats (SO, PO, RO, PKFD, PF, PL, and BT) decreased significantly.

**Table 3:** Effects of the dietary fat application of various broiler chicken diets on selected serum biochemical and physiological indices.

	Treatment								^9^SEM	*P* value
	^1^BSFL	^2^SO	^3^RO	^4^PO	^5^PKFD	^6^PF	^7^PL	^8^BT		
Biochemical indices										
Glucose, mg/dl	161	164	166	156	158	158	152	165	1.92	0.555
^10^TG, mg/dl	121	146	133	122	128	131	130	144	2.95	0.270
Cholesterol, mg/dl	185	193	192	179	177	184	173	182	3.89	0.896
Albumin, g/dl	2.21	2.75	2.50	2.44	2.40	2.60	2.53	2.47	0.05	0.053
^11^TP, g/dl	6.73	6.74	6.84	6.50	6.22	6.60	6.47	6.24	0.07	0.299
^12^ALT, IU/L	5.82	6.27	5.38	5.82	10.3	10.8	6.72	5.82	1.06	0.349
^13^AST, IU/L	73.3	74.1	68.9	68.4	58.8	56.8*	66.5	61.5	1.53	0.022
^14^ALP, IU/L	906	684	580	541*	347***	762	811	734	35.8	0.001
^15^GGT, IU/L	4.42	8.57	8.84	7.27	4.42	4.42	4.15	4.42	0.56	0.147
Immune indices										
^16^IgA, mg/ml	7.81	4.86*	5.65	5.01*	5.45	5.63	5.06*	6.36	0.20	0.022
^17^IgG, mg/ml	9.34	6.69	6.32	6.10*	6.68	7.67	7.11	10.20	0.33	0.007
^18^IgY, μg/ml	2172	2431	1968	1996	1550	1387	1146*	1950	91.1	0.002
^19^TNF-α, ng/l	264	248	237	289	188	186	169	231	18.3	0.426
^20^IL-6, ng/l	483	318	348	288	310	287	216**	309	19.6	0.008
Selected hormones										
^21^T3, nmol/l	7.99	6.93	6.67	5.37	5.42	5.84	5.26	7.20	0.32	0.168
^22^T4, nmol/l	111	68.0	81.4	75.5	68.0	64.0	68.5	82.6	3.75	0.059
^23^FT3, pmol/l	18.7	12.6*	12.4*	10.8**	12.6*	11.7**	10.0***	12.6*	0.58	< 0.001
^24^FT4, pmol/l	34.3	29.6	29.7	30.3	24.0	32.1	30.4	36.3	1.21	0.678
Insulin, IU/l	126	130	94	110	75	62	71	89	7.71	0.313

*p < 0.05, **p < 0.01, ***p < 0.001—the reference group (BSFL) is significantly different within a row when asterisks are present.

^1^BSFL—basal diet with 100% black soldier fly (*H. illucens*) larval fat; ^2^SO—basal diet with 100% soybean oil; ^3^RO—basal diet with 100% rapeseed oil; ^4^PO—basal diet with 100% palm oil; ^5^PKFD—basal diet with 100% palm kernel fat distillate; ^6^PF—basal diet with 100% poultry fat; ^7^PL—basal diet with 100% pig lard; ^8^BT—basal diet with 100% beef tallow; ^9^SEM—standard error of the mean; ^10^TG—triglyceride; ^11^TP—total protein; ^12^ALT—alanine transaminase; ^13^AST—aspartate transaminase; ^14^ALP—alkaline phosphatase; ^15^GGT—gamma-glutamyl transferase; ^16^IgA—immunoglobulin A; ^17^IgG—immunoglobulin G; ^18^IgY—immunoglobulin Y; ^19^TNF-α—tumor necrosis factor-alpha; ^20^IL-6—interleukin-6; ^21^T3—triiodothyronine; ^22^T4—thyroxine; ^23^FT3—free triiodothyronine; ^24^FT4—free thyroxine; Means represent 1 bird randomly chosen from each pen (*n* = 8).

Analysis of variance (ANOVA) or the Kruskal‒Wallis test, depending on the normal distribution of the data, provides the probability level (*P* value) presented in the last column.

### Cecal microbiota populations

The microbiota analysis revealed differences in the total number of microorganisms, as determined by 4’,6-diamidino-2-phenylindole (DAPI) staining, with the PF and PL groups exhibiting a notable increase (*P* = 0.047) compared with the BSFL group in terms of the overall microbiota count. However, all of the selected microbial populations, including the *Bacteroides‒Prevotella* cluster, *Clostridium leptum* subgroup, *Clostridium perfringens*, Enterobacteriaceae, *Clostridium coccoides‒Eubacterium rectale* cluster, and *Lactobacillus* sp./*Enterococcus* sp., showed no changes in numbers (*P* > 0.05) between broilers fed BSFL and those fed the other selected dietary fat sources (Supplementary Table S4).

### Carcass and meat quality traits

The administration of different dietary fats significantly affected carcass traits. Drumstick yield increased (*P* = 0.004) in animals fed BSFL compared with those fed BT, while thigh yield decreased (*P* = 0.010) in animals fed BSFL compared with those fed RO and PO (Supplementary Table S5). However, no differences (*P* > 0.05) were detected regarding other traits, such as final BW; carcass weight; and carcass, breast, leg quarter, wing, or giblet yields, among the broilers fed dietary fats relative to those fed BSFL.

The breast muscle pH in animals fed BT was lower immediately after slaughter (*P* = 0.002), 20 min (*P* < 0.001), and 2 h (*P* < 0.001) than that of broilers fed BSFL. The meat pH was lower (*P* < 0.001) in PF treatment compared to BSFL group only after 2 h (Table 4). However, no significant difference (*P* > 0.05) was recorded after 24 h in terms of the pH of the pectoralis majors in any group. The PF, PL, and BT treatments had reduced drip loss after 48 h (*P* = 0.021) than BSFL group. Furthermore, the treatments where animal fats were applied, along with those fed RO and PO, presented lower drip loss (*P* < 0.001) after 7 days than did the BSFL group. The PL group presented greater (*P* < 0.001) salt-induced water uptake (SALT) than did the BSFL group. Changes in the color of the breast meat showed that a* index decreased (*P* = 0.006) in the PL group, whereas the b* index increased (*P* = 0.007) in the BT group compared with the reference group. There were no changes (*P* > 0.05) in liver color.

**Table 4:** Effects of the application of various dietary fat sources to broiler chicken diets on the selected meat quality traits.

	Treatment								^9^SEM	*P* value
	^1^BSFL	^2^SO	^3^RO	^4^PO	^5^PKFD	^6^PF	^7^PL	^8^BT		
Breast meat										
pH _slaughter_	6.51	6.70	6.60	6.77	6.62	6.28	6.61	6.18*	0.04	0.002
pH _20 min_	6.40	6.48	6.34	6.38	6.26	6.23	6.26	6.06*	0.03	< 0.001
pH _2 h_	6.31	6.27	6.11	6.24	6.09	6.01**	6.15	5.98**	0.03	< 0.001
pH _24 h_	5.76	5.66	5.49	5.67	5.58	5.39	5.67	5.87	0.05	0.103
Drip loss _48 h_	4.65	3.85	3.85	3.84	3.68	3.09**	3.26**	3.42*	0.11	0.021
Drip loss _7 days_	14.0	12.1	10.9*	11.2*	12.3	10.6**	10.6**	10.4**	0.21	< 0.001
^10^SALT	15.2	12.0	10.2	13.7	17.6	17.6	27.8*	19.3	1.00	< 0.001
^11^L*	37.6	37.5	35.6	35.4	35.0	38.1	38.3	37.9	0.49	0.177
^12^a*	3.28	5.74	5.04	3.40	2.96	2.11	0.68*	1.57	0.38	0.006
^13^b*	4.84	5.44	4.24	5.01	7.46	5.33	5.76	10.32**	0.42	0.007
Liver										
^11^L*	20.7	22.0	20.8	20.7	23.1	24.5	22.1	19.9	0.58	0.309
^12^a*	6.09	6.90	9.61	6.19	5.61	4.22	3.98	3.19	0.49	0.079
^13^b*	6.15	6.82	6.94	4.48	9.12	7.22	7.08	11.06	0.54	0.216

*p < 0.05, **p < 0.01, ***p < 0.001—the reference group (BSFL) is significantly different within a row when asterisks are present.

^1^BSFL—basal diet with 100% black soldier fly (*H. illucens*) larval fat; ^2^SO—basal diet with 100% soybean oil; ^3^RO—basal diet with 100% rapeseed oil; ^4^PO—basal diet with 100% palm oil; ^5^PKFD—basal diet with 100% palm kernel fat distillate; ^6^PF—basal diet with 100% poultry fat; ^7^PL—basal diet with 100% pig lard; ^8^BT—basal diet with 100% beef tallow; ^9^SEM—standard error of the mean; ^10^Salt—salt-induced water uptake; ^11^ L*—lightness; ^12^a*—redness index; ^13^b*—yellowness index; Means represent 1 bird randomly chosen from each pen (*n* = 10).

Analysis of variance (ANOVA) or the Kruskal‒Wallis test, depending on the normal distribution of the data, provides the probability level (*P* value) presented in the last column.

### Breast meat and liver fatty acid profile

In contrast to the BSFL treatment, the SO group resulted in significantly less (*P* < 0.001) linolenic acid (C18:3 n3) and overall n-3 FAs in breast meat (Table 5). Unlike BSFL treatment, PKFD decreased PUFAs (*P* < 0.001) and increased the n6/n3 ratio (*P* = 0.002) and thrombogenic index (TI; *P* < 0.001). Compared with the BSFL group, the PL treatment showed significantly lower levels of palmitic acid (C16:0), saturated fatty acids (SFAs), atherogenic index (AI), and thrombogenic index (TI) (*P* < 0.001). Additionally, the PL group had significantly higher levels of oleic acid (C18:1 n9; *P* = 0.029), unsaturated fatty acids (UFAs; *P* < 0.001), monounsaturated fatty acids (MUFAs; *P* = 0.027), and the PUFA/SFA ratio (P < 0.001). Compared with BSFL, feeding PF increased (*P* < 0.001) SFA and TI levels, while decreasing (*P* < 0.001) UFA levels, PUFA levels, and the PUFA/SFA ratio.

**Table 5:** Effect of various dietary fat applications in broiler chicken diets on the breast muscle fatty acid profile (g/100 g FA).

Component	Treatment								^9^SEM	*P* value
	^1^HI	^2^SO	^3^RO	^4^PO	^5^PKFD	^6^PF	^7^PL	^8^BT		
Saturated fatty acids										
C12:0	0.18	0.18	0.19	0.19	0.18	0.18	0.18	0.18	0.001	0.733
C14:0	0.96	0.94	0.96	0.96	0.94	0.93	0.94	0.94	0.003	0.311
C15:0	0.15	0.14	0.14	0.14	0.14	0.13	0.14	0.13	0.001	0.128
C16:0	15.9	16.2	15.6	15.5	16.7	16.6	14.7*	16.1	0.116	< 0.001
C17:0	0.13	0.13	0.13	0.13	0.13	0.13	0.14	0.12	0.002	0.243
C18:0	4.93	5.08	4.87	5.31	4.92	5.30	5.08	5.05	0.068	0.662
C20:0	0.19	0.18	0.19	0.18	0.19	0.19	0.18	0.18	0.001	0.103
Unsaturated fatty acids										
C16:1 n7	1.39	1.39	1.42	1.41	1.40	1.39	1.38	1.41	0.007	0.775
C17:1	1.51	1.49	1.49	1.51	1.50	1.51	1.49	1.48	0.006	0.893
C18:1 n9	34.7	34.9	35.2	35.0	34.8	34.7	35.5*	34.5	0.071	0.029
C20:1	0.08	0.08	0.08	0.09	0.09	0.09	0.08	0.09	0.001	0.099
C18:2 n6	37.0	37.2	37.0	37.0	36.8	36.6	36.9	37.0	0.062	0.534
C18:3 n3	2.34	1.63*	2.27	2.24	1.76	1.71	2.86	2.28	0.071	< 0.001
C18:3 n6	0.34	0.35	0.35	0.34	0.35	0.35	0.34	0.34	0.001	0.606
C20:2	0.17	0.16	0.15	0.14	0.14	0.16	0.15	0.16	0.004	0.280
Summarized fatty acid										
^10^SFA	22.4	22.8	22.1	22.4	23.2	23.4*	21.3*	22.7	0.110	< 0.001
^11^UFA	77.6	77.2	77.9	77.6	76.8	76.6*	78.7*	77.3	0.110	< 0.001
^12^MUFA	37.7	37.9	38.2	38.0	37.8	37.7	38.4*	37.5	0.071	0.027
^13^PUFA	39.9	39.3	39.7	39.6	39.0*	38.9**	40.3	39.8	0.085	< 0.001
PUFA/SFA	1.78	1.72	1.80	1.77	1.69	1.66*	1.89*	1.76	0.012	< 0.001
n6	37.4	37.5	37.3	37.3	37.1	37.0	37.3	37.4	0.062	0.548
n3	2.34	1.63*	2.27	2.24	1.76	1.71	2.86	2.28	0.071	< 0.001
n6/n3	16.0	33.6	16.5	16.6	50.0*	23.8	13.8	16.5	3.198	0.002
^14^AI	0.257	0.262	0.252	0.252	0.269	0.268	0.237*	0.260	0.002	< 0.001
^15^TI	0.489	0.524	0.480	0.490	0.528*	0.538**	0.446*	0.499	0.005	< 0.001

*p < 0.05, **p < 0.01, ***p < 0.001—the reference group (BSFL) is significantly different within a row when asterisks are present. ^1^BSFL—basal diet with 100% black soldier fly (*H. illucens*) larval fat; ^2^SO—basal diet with 100% soybean oil; ^3^RO—basal diet with 100% rapeseed oil; ^4^PO—basal diet with 100% palm oil; ^5^PKFD—basal diet with 100% palm kernel fat distillate; ^6^PF—basal diet with 100% poultry fat; ^7^PL—basal diet with 100% pig lard; ^8^BT—basal diet with 100% beef tallow; ^9^SEM—standard error of the mean; ^10^SFA—saturated fatty acids; ^11^UFA—unsaturated fatty acids; ^12^MUFA—monounsaturated fatty acids; ^13^PUFA—polyunsaturated fatty acids; ^14^AI—atherogenic index; ^15^TI—thrombogenic index; Means represent 1 bird randomly chosen from each pen (*n* = 10).

Analysis of variance (ANOVA) or the Kruskal‒Wallis test, depending on the normal distribution of the data, provides the probability level (*P* value) exhibited in the last column.

Palmitic acid levels were lower (*P* < 0.001) in the BSFL group than in the SO, PO, or PF treatments, whereas palmitoleic acid levels were lower in the PKFD, PF, and PL groups in liver (Supplementary Table S6). Conversely, alpha-linolenic acid (ALA) and n-3 FA levels increased (*P* < 0.001) in the SO, PKFD, and PF groups compared to in the BSFL group. In terms of SFA levels, a decrease was seen in the SO and PF groups (*P* < 0.001), whereas they were increased in the PL group compared with the BSFL group. The UFA concentration increased (*P* < 0.001) in the SO and PF groups but decreased in the PL group relative to the BSFL group. Compared with those in the BSFL group, MUFA levels in the SO group increased (*P* < 0.001), but those in the PKFD group decreased. The PUFA concentration increased (*P* < 0.001) in the SO, PKFD, and PF groups relative to that in the BSFL treatment. The AI decreased (*P* < 0.001) in the SO, PO, and PF groups, whereas the TI decreased (*P* < 0.001) in the SO, PKFD, and PF groups in contrast to the BSFL group. Moreover, no significant changes (*P* > 0.05) were observed in the following SFAs (C14:0, C18:0, C20:0, and C22:0) and UFAs (C14:1, C18:1 n9, C18:2 n6, C18:3 n6, C20:2, and C20:4) between the experimental treatments and the reference group.

### Sensory analyses

The sensory traits of the broiler breast fillets are shown in Supplementary Table S7. The cooked odor of the broiler meat decreased (*P* = 0.008) in the PF and BT groups compared with that in the BSFL group. All the other sensory traits, including appearance, smell, texture, and taste parameters, were not different (*P* > 0.05).

## Discussion

To our knowledge, this is the first study to compare the characteristics of broilers fed BSFL fat with those fed a wide range of selected plant oils or animal fats, as previous research has focused primarily on the effects of replacing SO with BSFL fat (summarized by Benzertiha et al.^3^). This study confirmed that BSFL fat was rich in SFAs, particularly C12:0, followed by myristic and palmitic acids, with the lauric acid content influenced by the rearing substrate^15^. In this study, a mixture of vegetable and fruit waste (in accordance with the GMP + system) was used as a rearing substrate for *H. illucens* larvae. The primary feed materials included apples, carrots, potatoes, cabbage, and the wheat bran, the latter being added to achieve 25% susbtrate dry matter. The use of the diet in invertebrate production resulted in a lauric acid concentration of 41.1% in the larvae biomass. In contrast, oleic acid was the primary FA in BSFL when mitigating mussels, rapeseed cake, and shrimp waste were used as substrates, possibly due to differences in carbohydrate content^16^. Regarding the plant oils, SO was rich in PUFAs, RO and PKFD were rich in MUFAs, and PO had more SFAs than UFAs, which is consistent with the literature^17^. In contrast to our findings, previous research has shown that PKFD had higher amount of SFAs; however, the FA profile changed according to the temperature used during palm kernel oil distillation^18^. PF and PL contained more UFAs than SFAs, whereas BT had a slightly greater quantity of SFAs than UFAs, which agrees with the findings of a previous study^19^. While BSFL fat shares similarities with fats such as PO and animal fats in terms of SFA content, its distinct FA profile, especially its C12:0 level, sets it apart. These variations in FA composition provide a foundation for exploring the effects of BSFL in comparison with those of commonly used dietary fat sources on broiler performance, nutrient digestibility, liver histomorphology, selected physiological and immunological indices, cecal microbiota shifts, carcass yield, final product quality and, ultimately, consumer acceptance.

The performance results align with those of previous studies indicating that substituting SO with BSFL fat had no effect on BWG, FI or FCR^20,21^. However, compared with broilers fed BSFL, those fed PKFD had increased BWGs during the 1st week, which was supported by a reduced FCR during the same period. Despite the high SFA content of BSFL fat, the FCR of the broilers fed BSFL was lower than those fed fats rich in SFAs (PO, PF, BT), making it more similar to vegetable oils, which are known to support better growth performance and nutrient digestibility in broilers than animal fats^22^. This similarity between BSFL and plant oils can be attributed to the presence of MCFAs, which are absorbed more easily and can be directly used by enterocytes as an energy source, which is highly important in the first week when bile and lipase production is low^6,23^. The present study clearly shows that the administration of BSFL fat in broiler diets increased the diet AME_N_ value, in contrast to broilers fed BT and PL at 14 and 28 days of age. Moreover, compared with that of BT, BSFL feeding improved DM and CP retention on the 14th day. Additionally, feeding BSFL increased EE more than feeding SO did, possibly due to enhanced lipase activity^24^. However, no differences in pancreatic enzyme activity, nutrient digestibility or AIDE were observed between groups at the end of the experiment. These results are in line with those of the weights and lengths of GIT segments, in which PKFD treatment reduced jejunum weight, in contrast to BSFL treatment. Although this finding can be considered an exception, generally, BSFL treatment did not trigger alterations in selected organ or GIT weights or lengths in 35-day-old broilers.

Compared with plant-derived oils, animal-derived fats negatively affect the liver function of broilers via the accumulation of SFAs, leading to fatty liver and increased ALT and AST levels^22^. BSFL treatment resulted in higher AST and ALP levels than PF, PO, and PKFD did, possibly due to its lower UFA content, which might increase the demand for de novo lipogenesis in the liver, the main site for FA synthesis in broilers^25^. To date, BSFL treatment has not been found to impact levels of liver function markers such as AST and ALT, similar to SO^11^. Kierończyk et al.^26^ emphasized the positive effects on AST and cholesterol reduction in the plasma of partial or total SO replacement with BSFL fat in broiler diets. The liver fat concentration may be affected by the CIELab system values. The results of the liver colorimetric and histomorphological analyses were not significant, which is consistent with previous findings of a study in which SO administration was completely replaced with that of BSFL^27^. These results suggest that BSFL fat does not have any adverse effects on the liver.

In general, BSFL treatment significantly impacts immune status by increasing lymphocyte numbers, lysozyme activity, and survival rates while reducing energy expenditure on immune responses in poultry^28^. Dietary fats rich in UFAs are known to increase immune responses in broilers, improving disease resistance and antibody levels, whereas fats high in SFAs can impair immune function^29,30^. Moreover, studies have shown that dietary fat sources rich in UFAs can enhance the immune response in broilers, potentially increasing their abilities to resist diseases such as infectious bursal disease (IBD) and increasing antibody levels against pathogens^29,31^. The increase in IgA levels in broilers fed BSFL compared with those fed SO, PO, or PL was likely due to the immunomodulatory effects of C12:0 in BSFL fat^32^. The elevated IgA concentration in the BSFL group suggests a positive impact on mucosal immunity, a trait commonly associated with immune-modulating dietary components^33^. The increased IgG plasma level in the BSFL treatment was only noted relative to that in the PO group, reflecting the beneficial effects of BSFL fat. These results indicate that BSFL exhibits immunomodulating activities comparable to those observed in broilers fed dietary fats of plant origin, such as SO, RO, and PKFD.

Thyroid hormones play pivotal roles in nutrient and energy metabolism, as well as performance, especially under heat stress, by regulating body temperature in broilers. The notable increase in FT3 levels following BSFL consumption suggests potential alterations in the protein binding dynamics of thyroid hormone, leading to a greater release of bound T3. Research has indicated that higher levels of FT3 may be a sign of hyperthyroidism and could have negative implications for health^34^. However, total T3 levels (both bound and free) were not significant. Moreover, BSFL treatment tended to increase T4 levels more than treatment with plant oils or animal fats. Elevated T4 levels are often associated with improved growth performance due to increased energy and protein metabolism and increased insulin-like growth factor levels^35^. In contrast to our findings, BSFL treatment was found to decrease T4 and FT4 levels compared with SO treatment^36^. Nevertheless, elevated serum concentrations of T3 and T4 were also revealed in hens when fish meal was substituted with BSFL meal^37^. Furthermore, the high variability of the results was confirmed by a previous study^38^, in which it was reported that thyroid hormone levels vary significantly among experiments. In general, thyroid hormones are positively correlated with the growth of the bursa of Fabricius, the primary site for B-cell development in birds, which plays a role in humoral immunity via the production of antibodies^39^. However, further research is needed to fully understand the effect of BSFL fat on the humoral responses of birds.

BSFL showed no significant differences in cecal bacterial populations, except for a lower total bacterial count compared with those of the PL and PF groups. However, this significance is not physiologically or practically relevant because of the minimal variation in fat percentages. These findings align with previous research on the cecal microbiota of broilers when BSFL was replaced with SO^21,26^. However, the lack of significance results in broilers fed BSFL compared with those fed SFA-rich fat sources was unexpected given the role of SFAs in promoting the growth of lipopolysaccharide-producing bacteria and pathogenic bacteria such as *C. perfringens* and lactic acid-producing bacteria reported in previous studies^30,40^. Moreover, the lower total microorganism count in the BSFL treatment than in the PF and PL groups is difficult to explain, particularly when broilers fed the other dietary fats, i.e., those of plant or animal origin, exhibited no changes. Thus, the obtained results cannot be associated with high or low concentrations of UFAs or SFAs.

BSFL treatment showed minimal differences in broiler carcass yield (CY), part yield (PY), and giblet yield (GY) compared to plant and animal fat sources, except for drumstick and thigh yields. Diets with plant oils typically result in better carcass quality than diets including animal fats such as tallow or lard, which tend to increase lipid accumulation^41,42^. The improved drumstick yield in the BSFL group compared with the BT group may be due to the long-chained SFAs in BT, as BT treatment increased fat deposition in the abdomen instead of in meat compared with than in animals fed plant oils, which was related to differences in fatty acid composition. Compared with long-chain FAs, which can affect broiler growth, MCFAs are generally more easily absorbed by enterocytes and are more digestible^6^ compared to long-chain FAs, which can affect broiler growth. Moreover, SFAs promote abdominal fat deposition rather than muscle tissue development^43^. However, compared with the other treatments, the BSFL treatment did not affect CY, PY, or GY. Interestingly, the thigh yield for the BSFL group was lower than that for the PO and RO groups. These results suggest differences, but further research is needed. Previous studies have shown that replacing SO with BSFL had no effect on the thigh, breast, or wing mass (% of CW) of poultry^10,27,44^. Consistent with these findings, our study also revealed no effect of BSFL on the pH of broiler meat relative to the SO group^14,27,44^. BSFL treatment increased the meat pH compared with BT (at slaughter, 20 min, and 2 h after slaughter) and PF (2 h after slaughter), likely due to lactic acid accumulation from muscle glycolysis^45^. No significant difference in meat pH was observed between the BSFL treatment and other groups after 24 h. In addition to pH, the effect of BSFL on meat color, drip loss and salt uptake was also evaluated. Regarding meat color, our findings align with a previous study, which also reported no significant effect on the L*, a*, or b* values when substituting SO with BSFL^46^. However, contrasting results were found in another study, where BSFL treatment reduced meat redness and increased yellowness compared with SO^14^. Furthermore, the difference in a* and b* between broilers fed BSFL and those fed PL or BT could be attributed to oxidative changes induced by animal fats, affecting meat color via myoglobin interactions^47^. Although lower meat pH and greater lightness are commonly associated with increased drip loss and reduced salt-induced water uptake^48^, the differences in drip loss and salt-induced water uptake observed in this study were an exception, with no difference in pH and yellowness between BSFL and these fat sources. Additionally, our results with SO were inconsistent with those of previous studies, which reported reduced drip loss and increased SALT when BSFL replaced SO^14^.

The FA composition of the breast meat of broilers fed BSFL did not align with the FA composition of the experimental diet. Despite BSFL being high in SFAs, breast meat presented higher levels of UFAs than SFAs, ultimately changing the overall UFA/SFA ratio. This finding contrasts with previous findings in which BSFL decreased PUFAs while increasing SFAs and MUFAs in poultry meat^44,46^. However, another study supports our results, showing that including more BSFL (up to 9%) in the diet can lead to increased UFAs rather than increased SFAs and PUFA/SFA ratios^14^. However, the increased ALA and n-3 FA contents in broilers fed BSFL compared with those fed SO were unexpected, as previous studies had reported that BSFL negatively affects the presence of n-3 FAs in meat^12,14^. Furthermore, the TI and AI indices, which are important for assessing cardiovascular health, were similar to those of birds fed plant oils and aligned with studies in which BSFL treatment replaced that of SO in poultry diets^14,44^. The liver FA profiles of the broilers fed BSFL differed from those of broilers fed plant oils (SO, PKFD, PO) or animal fats (PF, PL), which is consistent with previous findings in which BSFL treatment reduced abundance of UFAs, especially of n-3, and increased the abundance of SFAs when used to replace SO treatment^21^. Although the liver is a site of de novo synthesis^25^, we observed a lower concentration of n-3 FAs in the liver compared to the breast muscle, which suggests that n-3 FAs were transported through the bloodstream, and was incorporated into the breast meat. Consumer acceptance of BSFL in poultry nutrition relies heavily on the sensory traits of the final products, such as flavor, texture, and appearance, which are crucial for marketability. Plant oils, which are rich in UFAs, generally increase the sensory appeal of poultry meat, making it more acceptable than meat from broilers fed animal fats. However, higher n-3 and PUFA levels can negatively impact taste and storage quality due to increased oxidation risks^49^. The increased odor in BSFL treatment compared with PF or BT group could be due to the oxidation of UFAs to volatile organic compounds (VOCs) during cooking^50^. This aligns with our findings that breast meat from BSFL-fed broilers had a higher UFA content than did that from birds fed animal fat. Nevertheless, other sensory traits, such as texture, taste, color, and smell, remained unaffected. Moreover, our results are consistent with those of previous experiments in which birds fed BSFL fat presented a sensory profile similar to that of birds fed SO^12,46^. This finding is supported by the FA composition of breast meat, in which UFAs were more abundant in the muscles of the broilers fed BSFL than in those fed animal fats.

In conclusion, this study demonstrated that the inclusion of BSFL fat in broiler diets results in outcomes closely resembling those of plant oils rather than animal fats in terms of growth performance, cecal microecosystem, meat quality, and sensory attributes. Surprisingly, BSFL-fed birds presented greater levels of n-3 FAs and alpha-linolenic acid in their breast meat than SO-fed broilers did, improving their nutritional profile. However, more focus should be placed on FA profile of *H. illucens* larvae. Additionally, our findings revealed that, compared with that of plant oils (SO and PO), BSFL treatment also increased IgA or IgG levels, indicating an improved immune response. Furthermore, insect fat may tend to induce a positive increase in thyroid hormone levels in bird plasma. Overall, *H. illucens* larval fat did not adversely affect carcass traits except for thigh yield, which was greater in RO and PO. These results suggest the promising potential of BSFL fat as a sustainable feed ingredient.

## Methods

### Birds and housing

One thousand two hundred 1-day-old male Ross 308 chicks (initial weight 40 g ± 0.5 g) were obtained from a commercial hatchery. The birds were randomly divided into eight dietary groups comprising 10 replicate pens and 15 birds (for 14 days) or 10 birds (for 35 days) per pen. The broilers were housed in 1 m × 1 m floor pens with chopped wheat straw (7–15 cm) as litter. The experiment was conducted in a broiler house (number 0161) located in Olszowa, Poland. To simulate intensive conditions, the pens were surrounded by 9000 unsexed broilers of the same origin. The greater number of birds observed at 14 days was related to the need for enough excreta sampling for further nutrient retention examination. After 2 weeks, 5 randomly chosen broilers per pen were excluded from the experiments to obtain the correct animal density in the replicate pen and were transferred to the main flock. The housing conditions were in accordance with the Aviagen: Ross Broiler Management Handbook^51^ and the Council Directive (2007/43/EC)^52^. The broilers were vaccinated in a hatchery against infectious bronchitis (Nobilis IB Ma5 and Nobilis IB 4/91; Intervet International B.V., Boxmeer, Netherlands). On days 12 and 20 after hatching, the chicks were vaccinated against Gumboro disease (AviPro PRECISE, Lohmann Animal GmbH, Cuxhave, Germany).

### Diets

The compositions and nutritive values of the experimental diets are detailed in Tables 6 and 7. The study involved eight experimental diets over two dietary periods, i.e., starter (up to 14 days) and grower (from 15 to 35 days). This study was designed to enrich basal diets with selected plant oils and animal fats such as BSFL fat, soybean oil (SO), rapeseed oil (RO), palm oil (PO), palm kernel fatty acid distillate (PKFD), poultry fat (PF), pig lard (PL), or beef tallow (BT). BSFL fat was purchased from HiProMine S.A. (Robakowo, Poland), while the other dietary fats were obtained from Piast Pasze Sp. z o.o. (Lewkowiec, Poland). The formulation of the diets was conducted in accordance with ISO 9001:2008 guidelines. The diets were optimized to meet or exceed the nutrient requirements recommended by Aviagen for broilers^53^. The diets were isonitrogenous and isoenergetic and offered from 1 to 35 days of age. The apparent metabolizable energy (AME) level of BSFL fat for broilers was established according to the regression model proposed by Kierończyk et al.^4^. The AMEs of other dietary fats were adapted from INREA-CIRAD-AFZ (2021)^54^. The conditions for insect production and dietary fat extraction were maintained as previously described by Kierończyk et al.^24^. Using a laboratory-scale line, the diets were processed through roller mills (Skiold, Sæby, Denmark) and a horizontal double band mixer (Zuptor, Gostyń, Poland). The feeds were subsequently prepared in mashed form by grinding all the raw materials via a disc mill (Skiold A/S, Denmark) at a 2.5 mm disc distance. This mixture was blended without heat treatment and was free of any additional additive inclusions, such as exogenous enzymes or coccidiostats. Throughout the entire experimental period, broilers were provided ad libitum access to feed (one hopper per pen) and water (two nipple drinkers per pen).

**Table 6:** Nutrient compositions (g/kg) of starter diets administered to broiler chickens up to 14 days of age.

Ingredients, g kg^−1^	^1^BSFL	^2^SO	^3^RO	^4^PO	^5^PKFD	^6^PF	^7^PL	^8^BT
Maize	554.5	554.5	554.5	532.4	550.1	553.2	548.2	535.4
Soybean meal, 468 g kg^−**1**^	369.7	369.7	369.7	373.2	370.4	369.9	370.7	372.8
Dietary fat^a^	34.0	34.0	34.0	52.6	37.7	35.1	39.3	50.1
Vitamin premix^b^	3.00	3.00	3.00	3.00	3.00	3.00	3.00	3.00
Dicalcium phosphate	18.7	18.7	18.7	18.8	18.7	18.7	18.7	18.8
Limestone	10.1	10.1	10.1	10.0	10.1	10.1	10.1	10.0
NaCl	2.35	2.35	2.35	2.38	2.40	2.35	2.36	2.38
Na_2_SO_4_	1.48	1.48	1.48	1.44	1.50	1.48	1.47	1.45
L-Lysine	1.98	1.98	1.98	1.92	2.00	1.97	1.96	1.92
L-Methionine	2.81	2.81	2.81	2.83	2.80	2.81	2.81	2.83
L-Threonine	0.99	0.99	0.99	0.98	1.00	0.99	0.99	0.98
L-Valine	0.42	0.42	0.42	0.42	0.40	0.42	0.42	0.42
Calculated nutritive value, g kg^−1^								
AME_N_, kcal kg^−**1**^	3000	3000	3000	3000	3000	3000	3000	3000
Crude protein	215.5	215.5	215.5	215.5	215.5	215.5	215.5	215.5
Crude fat	59.9	59.9	59.9	77.6	63.4	60.9	65.0	75.3
Crude fiber	26.6	26.6	26.6	26.3	26.6	26.6	26.5	26.3
Calcium	8.5	8.5	8.5	8.5	8.5	8.5	8.5	8.5
Lysine	13.1	13.1	13.1	13.1	13.1	13.1	13.1	13.1
Methionine + Cystine	9.8	9.8	9.8	9.8	9.7	9.8	9.7	9.8

^a^The following dietary fats were added: ^1^BSFL—*H. illucens* larval fat; ^2^SO—soybean oil; ^3^RO—rapeseed oil; ^4^PO—palm oil; ^5^PKFD—palm kernel fat distillate; ^6^PF—poultry fat; ^7^PL—pork lard; ^8^BT—beef tallow; ^b^Provided the following per kilogram of diet: vitamin A, 11 000 IU; cholecalciferol, 2 500 IU; vitamin E, 50 mg; menadione, 2.50 mg; vitamin B, 0.02 mg; folic acid, 1.0 mg; choline, 300 mg; D-pantothenic acid, 13.6 mg; riboflavin, 7.0 mg; niacin, 41.7 mg; thiamine, 2.0 mg; D-biotin, 0.20 mg; pyridoxine, 4.0 mg; ethoxyquin, 0.1 mg; Mn (MnO_2_), 60 mg; Zn (ZnO), 95 mg; Fe (FeSO_4_), 45 mg; Cu (CuSO_4_), 20 mg; I (CaI_2_O_6_), 0.6 mg; and Se (Na_2_SeO_3_), 0.35 mg.

**Table 7:** Nutrient compositions (g/kg) of grower diets administered to broiler chickens from 15 to 35 days of age.

Ingredients, g kg^−1^	^1^BSFL	^2^SO	^3^RO	^4^PO	^5^PKFD	^6^PF	^7^PL	^8^BT
Maize	629.2	629.2	629.2	595.7	622.5	627.1	619.6	600.2
Soybean meal, 468 g kg^−**1**^	283.8	283.8	283.8	289.0	284.9	284.2	285.3	288.3
Dietary fat^a^	51.5	51.5	51.5	79.70	57.1	53.2	59.5	75.9
Vitamin premix^b^	3.00	3.00	3.00	3.00	3.00	3.00	3.00	3.00
Dicalcium phosphate	8.58	8.58	8.58	8.65	8.60	8.58	8.60	8.64
Limestone	9.40	9.40	9.40	9.30	9.40	9.40	9.40	9.40
NaCl	1.49	1.49	1.49	1.51	1.50	1.49	1.50	1.51
Na_2_SO_4_	2.10	2.10	2.10	2.07	2.10	2.1	2.09	2.08
L-Lysine	5.00	5.00	5.00	5.00	5.00	5.00	5.00	5.00
L-Methionine	2.59	2.59	2.59	2.62	2.60	2.59	2.60	2.62
L-Threonine	2.82	2.82	2.82	2.89	2.80	2.83	2.84	2.88
L-Valine	0.53	0.53	0.53	0.54	0.50	0.53	0.53	0.53
Calculated nutritive value, g kg^−1^								
AME_N_, kcal kg^−1^	3200	3200	3200	3200	3200	3200	3200	3200
Crude protein	185.0	185.0	185.0	185.0	185.0	185.0	185.0	185.0
Crude fat	78.8	78.8	78.8	105.7	84.1	80.4	86.4	102.1
Crude fiber	24.9	24.9	24.9	24.4	24.8	24.9	24.8	24.5
Calcium	6.5	6.5	6.5	6.5	6.5	6.5	6.5	6.5
Lysine	13.3	13.3	13.3	13.3	13.3	13.3	13.3	13.3
Methionine + Cystine	8.6	8.6	8.6	8.6	8.6	8.6	8.6	8.6
Analyzed nutritive value, g kg^−1^								
Dry matter	874.9	874.8	874	874.7	872.1	881.2	874.5	873.7
Crude protein	180.6	181.5	182.4	182.5	178.8	184.5	187.7	183
Ether extract	77.5	75	75.2	73.6	78.7	73.5	77.4	75.8
Crude Fiber	28.4	25	28.8	28.7	27.6	24.8	29	29.4
Gross energy, kcal kg^−1^	4227	4135	4153	4191	4108	4201	4193	4086

^a^The following dietary fats were added: ^1^BSFL—*H. illucens* larvae fat; ^2^SO—soybean oil; ^3^RO—rapeseed oil; ^4^PO—palm oil; ^5^PKFD—palm kernel distillate; ^6^PF—poultry fat; ^7^PL—pork lard; ^8^BT—beef tallow; ^b^ Provided the following per kilogram of diet: vitamin A, 11 000 IU; cholecalciferol, 2 500 IU; vitamin E, 50 mg; menadione, 2.50 mg; vitamin B, 0.02 mg; folic acid, 1.0 mg; choline, 300 mg; D- pantothenic acid, 13.6 mg; riboflavin, 7.0 mg; niacin, 41.7 mg; thiamine, 2.0 mg; D-biotin, 0.20 mg; pyridoxine, 4.0 mg; ethoxyquin, 0.1 mg; Mn (MnO_2_), 60 mg; Zn (ZnO), 95 mg; Fe (FeSO_4_), 45 mg; Cu (CuSO_4_), 20 mg; I (CaI_2_O_6_), 0.6 mg; and Se (Na_2_SeO_3_), 0.35 mg.

### FA composition of the diets

The BSFL fat presented a high abundance of saturated fatty acids (SFAs), particularly C12:0, which comprised 41.1% of the total fatty acids (Table 8). Soybean oil (SO) significantly contributed to the abundance of polyunsaturated fatty acids (PUFAs), with linoleic acid (C18:2 n6) being a prominent constituent. Rapeseed oil (RO), palm kernel fatty acid distillate (PKFD), poultry fat (PF) and pig lard (PL) presented a prevalence of monounsaturated fatty acids (MUFAs), notably oleic acid (C18:1). Palm oil (PO) and beef tallow (BT) demonstrated a balance between SFAs and unsaturated fatty acids (UFAs), with significant amounts of palmitic acid (C16:0) and oleic acid (C18:1). Moreover, both PL and BT presented elevated levels of stearic acid (C18:0) compared with the other fat sources.

**Table 8:** Fatty acid compositions of the experimental diets.

Component	^1^BSFL	^2^SO	^3^RO	^4^PO	^5^PKFD	^6^PF	^7^PL	^8^BT
Saturated fatty acids								
C8:0 Caprylic acid	0.06	–	–	–	–	0.03	–	0.02
C10:0 Capric acid	1.23	–	–	–	–	–	–	–
C12:0 Lauric acid	41.1	–	–	0.2	–	0.04	–	0.13
C14:0 Myristic acid	15.91	0.1	0.02	1.1	–	1.0	1.22	6.34
C15:0 Pentadecanoic acid	–	0.01	–	–	–	0.1	–	0.51
C16:0 Palmitic acid	14.77	11.2	3.49	44.7	14.23	24.5	23.42	27.01
C17:0 Heptadecanoic acid	0.15	–	0.02	–	0.09	0.13	0.18	0.86
C18:0 Stearic acid	2.05	5	1.89	4.2	3.5	4.9	12.01	16.8
C20:0 Arachidic acid	0.1	0.21	0.5	0.1	0.58	0.06	–	–
C21:0 Heneicosanoic acid	0.21	–	–	–	–	–	–	–
C22:0 Docosanoic acid	0.05	0.25	0.24	–	–	0.04	–	0.03
C24:0 Tetracosanoic acid	–	0.05	0.11	–	–	–	–	–
Monounsaturated fatty acids								
C14:1 Myristoleic acid	0.2	–	–	–	–	0.11	–	–
C16:1 Palmitoleic acid	2.18	0.1	0.22	–	1.6	5.43	2.1	5.42
C17:1 Heptadecenoic acid	0.1	–	0.05	–	0.11	–	0.26	–
C18:1 Oleic acid	17.3	21.1	73.88	39.3	59.89	39.21	42.13	34.82
C20:1 Eicosenoic acid	–	0.1	1.4	–	–	0.1	0.68	–
C22:1 Erucic acid	–	–	0.67	–	–	–	–	–
C24:1 Nervonic acid	–	–	0.12	–	–	–	–	–
Polyunsaturated fatty acids								
C18:2 n6 Linoleic acid	1.36	52.28	11.27	10	16.61	21.81	16.08	1.55
C18:3 n3 Linolenic acid	0.3	7.5	1.23	0.1	0.23	0.3	1.33	–
C18:3 n6 y-linolenic acid	2.17	2.1	4.85	0.3	3.16	2	–	6.2
C18:4 n-3 Stearidonic acid	0.04	–	–	–	–	0.04	–	–
C20:5 n-3 Eicosapentaenoic acid	0.72	–	–	–	–	0.07	–	–
C20:2 Eicosadienoic acid	–	–	0.04	–	–	0.13	0.59	0.31
Summarized fatty acids								
^9^SFA	75.63	16.82	6.27	50.3	18.4	30.8	36.83	51.7
^10^UFA	24.37	83.18	93.73	49.7	81.6	69.2	63.17	48.3
^11^MUFA	19.78	21.3	76.34	39.3	61.6	44.85	45.17	40.24
^12^PUFA	4.59	61.88	17.39	10.4	20	24.35	18	8.06
PUFA/SFA	0.06	3.68	2.77	0.21	1.09	0.79	0.49	0.16

^1^BSFL—*H. illucens* larvae fat; ^2^SO—soybean oil; ^3^ RO—rapeseed oil; ^4^ PO—palm oil; ^5^ PKFD—palm kernel distillate; ^6^ PF—poultry fat; ^7^ PL—pork lard; ^8^ BT—beef tallow; ^9^ SFA—saturated fatty acids; ^10^ UFA—unsaturated fatty acids; ^11^ MUFA—monounsaturated fatty acids; ^12^ PUFA—polyunsaturated fatty acids.

### Data and sample collection

Throughout the trial (1–35 days), the body weights (BWs) and feed intakes (FIs) were monitored, and the body weight gains (BWGs) or feed conversion ratio (FCR) was calculated for the following periods: 1–7 days, 8–14 days, 15–21 days, 22–28 days, 29–35 days, and 1–35 days. The abovementioned traits were determined using a laboratory scale (NVL5101, OHAUS, Switzerland) with an accuracy of ± 0.5 g. For the growth performance results, the pen was defined as an experimental unit (*n* = 10). The excreta samples (1 pooled sample from 2 pens with 10 birds each, *n* = 5) were collected on days 14 and 28 in containers and immediately frozen on dry ice for further chemical analyses, nutrient retention and apparent metabolizable energy corrected to zero nitrogen balance (AME_N_) calculations, described in detail in the following subheadings. During sample collection, the plastic mat at the bottom of the pen was unfolded to prevent contamination with feed, feathers, and litter. At the end of the experiment (35th day), a total of 80 broilers (1 randomly chosen broiler from each replicate pen, *n* = 10) underwent electrical stunning (STZ-6, PPHU KOMA, Poland), followed by a sequence of slaughtering (cervical dislocation), exsanguination, dissection, and evisceration. Blood was drawn shortly after decapitation, while serum was obtained by centrifugation (Micro 220 R, Hettich, Tuttlingen, Germany) at 1000×*g* and 8 °C for 10 min and stored at − 20 °C until further analysis. A laboratory balance (LPC-523i, VWR International, Belgium; accurate to ± 0.001 g) was used to determine the weight (% of BW) of empty selected gastrointestinal tract (GIT) segments and internal organs (proventriculus, gizzard, duodenum, jejunum, ileum, ceca, heart, liver, pancreas, spleen, bursa of Fabricius, and thymus), and a linear scale accurate to 1 mm was used to measure the lengths of GIT segments (cm kg^−1^ of BW) in relation to live body weights. The duodenal digesta samples were collected into Eppendorf tubes (8 randomly selected broilers per treatment,* n* = 8) and placed immediately on dry ice for further determination of activities of pancreatic enzymes (trypsin, lipase, and amylase). The jejunal section began at the end of the duodenum and ended at Meckel’s diverticulum. The ileum was defined as the small intestinal segment caudal to Meckel’s diverticulum. Liver samples were fixed by immersion in Bouin solution and stored at 4 °C for further histomorphological analyses. Furthermore, the cecal digesta samples were gently squeezed into segments directly into Eppendorf tubes (1 bird per pen, 10 broilers per treatment, *n* = 10) and directly frozen. The remaining portions of the ileal contents from two birds (n = 5) were pooled and immediately frozen on dry ice to estimate the coefficients of apparent ileal digestibility (CAID) and apparent ileal digestible energy (AIDE). On the 35th day, the body weights at slaughter were measured (1 broiler from each replicate pen, *n* = 10). After bleeding, the birds were placed in a scalding tank (approximately 2 min, 60 °C; Technologies 4All Group, Kępno, Poland) and defeathered via an automatic turning machine (Technologies 4All Group, Kępno, Poland). Next, the carcass weights (CWs) were determined by weighing the entire slaughtered bird, both before and after evisceration, encompassing the neck and feet but excluding the head. Afterward, the carcasses were dissected into the following parts: breasts, leg quarters, drumsticks, thighs, and wings, and then weighed using a laboratory balance (NVL5101, OHAUS, Switzerland; accurate to ± 0.5 g). The carcass yield (CY), which represents the ratio of CW to body weight at slaughter, was calculated. The parts yield (PY), including the breast, leg quarters, drumsticks, thighs, and wings, was determined as the ratio of each selected part weight to the CW. The giblet yield (GY), consisting of heart, clean and empty gizzards, and liver, was calculated as the percentage of giblet weight (GW) relative to CW, with GW representing the sum of giblet weights. The breast meat samples and liver samples were sealed in flex–grip bags, immediately placed on dry ice, and stored at − 20 °C for further chemical analyses.

### Chemical analysis

The dry matter (DM), crude protein (CP), ether extract (EE), and amino acid contents of the experimental feed, excreta and ileal digesta were examined via the 934.01, 976.05, 920.39, and 994.12 (AOAC) methods, respectively^55^. The DM, CP, and EE analyses were performed via an ED-56 air drier (Binder 9010–0333, Tuttlingen, Germany), a Kjel-Foss automatic 16 210 analyzer (A/S N. Foss Electric, Hillerød, Denmark), and a Soxtec System HT-1043 extraction unit (Foss Tecator, Hillerød, Denmark), respectively. The gross energy (GE) was measured via an adiabatic bomb calorimeter (KL 12 Mn, Precyzja-Bit PPHU, Bydgoszcz, Poland) standardized with benzoic acid (Avantor Performance Materials Poland S.A., Gliwice, Poland). Furthermore, titanium dioxide (TiO_2_) was added ‘on top’ of the experimental diets and used as an internal marker to estimate the digestibility of nutrients. The sample preparation and estimation of the TiO_2_ concentration were performed as described by Myers et al.^56^ and Short et al.^57^. The FA profiles of the experimental diets, breast muscles, and liver tissues were analyzed via the methods outlined by Stuper-Szablewska et al.^58^.

### Histomorphological examination

Histological analysis of the hepatic tissues was performed as described by Rawski et al.^59^. Briefly, broiler chicken liver samples were preserved by immersion in Bouin’s solution and storing at 4 °C for 24 h. Liver samples were subjected to histological examination using the paraffin method and were stained with hematoxylin and eosin (H&E). A semiquantitative scoring system was used to assess the severity of histopathological changes, considering factors such as parenchymal eclipse, the presence of vacuoles in hepatocytes, the number of congestions, necrosis, fibrosis, and the amount of fat vacuolization. The scoring system employed a 5-point (0–4) scale: 0 indicated no changes, 1 indicated slight histopathology in less than 25% of the fields, 2 represented mild histopathology in less than 50% of the fields, 3 signified moderate histopathology in less than 75% of the fields, and 4 indicated severe histopathology in more than 75% of the fields.

### Selected blood serum parameters and pancreatic enzymes

Selected serum parameters, including glucose, triglyceride (TG), total cholesterol, total protein, albumin, aspartate transaminase (AST), alkaline phosphatase (ALP), gamma-glutamyl transferase (GGT), and alanine transaminase (ALT) levels, were assessed following the manufacturer’s instructions for commercially available kits (Pointe Scientific, Warsaw, Poland). The immunological indices, i.e., immunoglobulin Y (IgY), immunoglobulin A (IgA), immunoglobulin G (IgG), interleukin-6 (IL-6), and tumor necrosis factor-alpha (TNF-α) concentrations, as well as levels of selected metabolic hormones, i.e., insulin, triiodothyronine (T3), thyroxine (T4), free triiodothyronine (FT3) and free thyroxine (FT4), were determined in the collected plasma on the basis of the manufacturer’s instructions for available commercial kit reagents (Shanghai Sunred Biological Technology Co., Ltd., Shanghai, China or Abnova Corporation, Taipei, Taiwan). For the absorbance measurements, a Synergy 2 multidetection microplate reader (Biotek Instruments, Inc., Winooski, Vt., USA) was used. The endogenous enzyme activities were measured via commercially available colorimetric assay kits (BioVision, Milpitas, CA, USA) as described by Kierończyk et al.^60^.

### Cecal microbiota populations

All details of sample preparation and fluorescence in situ hybridization (FISH) analyses for bacterial enumeration from the cecal digesta were described by Sypniewski et al.^61^. In brief, 100 μL of digesta was diluted in PBS, transferred onto 0.22 µm polycarbonate filters (Frisenette, Knebel, Denmark), and subjected to vacuum filtration (Vacuum Pump, KNF Neuberger, Trenton, NJ, USA). The filters were then placed on cellulose discs and dehydrated in ethanol. Hybridization was performed in 50 μL of hybridization buffer (0.9 M NaCl, 20 mM Tris/HCl, pH 7.2, 0.01% SDS) containing the oligonucleotide probes (Supplementary Table 8). The filters were subsequently washed with washing buffer (20 mM Tris/HCl, pH 7.2, 0.01% SDS, and 5 mM EDTA) for 20 min at 48 °C, rinsed with distilled water, dried, and mounted on slides with VectaShield (Vector Laboratories, Burlingame, CA, USA) anti-fading agent containing 4′,6-diamidino-2-phenylindole (DAPI). To differentiate the total bacterial count from other particles in the samples, the filters were kept at 4 °C in the dark for 1 h before visualization via an Axio Imager M2 (Carl Zeiss, Oberkochen, Germany).

### Carcass and meat quality traits

Postmortem (at slaughter, after 20 min, 2 h, and 24 h) pH measurements were taken via a ScienceLine Micro pH combination electrode N 6000 BNC (Schott SI Analytics, Mainz, Germany) inserted into the cranial end of the intact breast meat fillet. The pH meter used was a 1100 H pH meter (VWR International, Leuven, Belgium) with a ScienceLine Micro pH combination electrode N 6000 BNC (Schott SI Analytics, Mainz, Germany). Duplicate measurements were taken, and the mean value was used. Color measurements were performed according to the CIELab system^62^. The meat color analysis was performed at room temperature (20 °C), and the device was placed on the samples in a horizontal position. The color was measured three times on the internal side of each raw pectoral muscle or liver (*lobus dexter*) external side, and the mean of the triplicate determinations was used as an experimental unit (*n* = 10). Drip loss and salt-induced water uptake (SALT) were measured following the procedure described by Kierończyk et al.^14^. The evaluation of selected sensory attributes of the broiler chicken breast fillets was performed in accordance with the methods of Altmann et al.^63^. The color intensity, smell (overall odor, animal/barn odor, metallic odor, and cooked chicken odor), texture (juiciness, hardness, adhesiveness), and taste (bitter taste, sour taste, sweet taste, metallic flavor, and chicken flavor) were measured via a 5-point scale. The scale ranged from 0 to 5 for visual attributes, such as color, and from 1 to 5 for olfactory, textural, and taste attributes.

### Calculations

The calculation of total tract nitrogen retention (NR) and CAID for ether extract (EE) was performed via the internal marker technique, which involves comparing values with TiO_2_ as a reference via the following formula:

$$CAID_{Nutrient} = 1 - \left\{ {\left( {\frac{{TiO_{2} \frac{g}{kg}diet}}{{TiO_{2} \frac{g}{kg}digesta}}} \right) \times \left( {\frac{{Crude Protein \frac{g}{kg}digesta}}{{Crude Protein \frac{g}{kg}diet}}} \right)} \right\}$$

On the basis of the GEs of the feed and ileal digesta, the AIDE was computed via the following equation:

$$AIDE = GE_{diet} - \left( {GE_{ileal digesta} \times \frac{{TiO_{2 diet} }}{{TiO_{2 ileal digesta} }}} \right)$$

An adjustment for zero nitrogen balance (8.22 kcal/N retained) was applied^64^, and the following formula was used to measure the AME_N_ of the experimental diets or excreta:

$$AME_{N} = \left\{ {GE_{diet} - \left( {GE_{excreta} \times \frac{{TiO_{2} diet}}{{TiO_{2} excreta}} } \right)} \right\} - 8.22 \times \left\{ {N_{diet} - \left( {N_{excreta} \times \frac{{TiO_{2} diet}}{{TiO_{2} excreta}} } \right)} \right\}$$

Using FA composition data, the following formulas were used to derive the atherogenic index (AI) and thrombogenic index (TI)^14^:

$$Atherogenic index = \left[ {\frac{{{\text{C}}12:{ }0{ } + { }4{\text{ x C}}14:{ }0{ } + {\text{ C}}16:{ }0}}{{\sum { }MUFA{ } + { }\sum \left( {n - 6} \right){ } + { }\sum \left( {n - 3} \right)}}} \right]$$

$$Thrombogenic index = \left[ {\frac{{C14:{ }0{ } + { }C16:{ }0{ } + { }C18:{ }0}}{{0.5 x \sum MUFA + 0.5 x \sum \left( {n - 6} \right) + 3 x \sum \left( {n - 3} \right) + \frac{{\sum \left( {n - 3} \right)}}{{\sum \left( {n - 6} \right)}}}}} \right]$$

### Statistical analysis

The experimental design employed complete randomization, designating the replicate pen (*n* = 10) as the experimental unit for growth performance results. In contrast, for various parameters, such as selected organ measurements, liver histomorphology, meat quality, and sensory traits, the experimental unit comprised 10 broilers, specifically one broiler randomly selected from each replicate pen (*n* = 10). Notably, CAID or nutrient retention determination for DM, CP, EE, AIDE, and AME_N_ utilized pooled digesta from two randomly chosen birds per replicate as the experimental unit (*n* = 5). For the biochemical and physiological serum indices as well as selected pancreatic enzymes, 8 randomly chosen broilers from each treatment were used (*n* = 8). Microbiota analyses were performed via digesta samples pooled from two broilers, and five photos were taken during microscopy. The mean bacterial count obtained from five photos was defined as the experimental factor (*n* = 5). RStudio (ver. 2024.04.0 + 735 RStudio, Inc., Boston, USA) facilitated the statistical analyses with the following packages: *FSA* (ver. 0.9.4), *DescTools* (ver. 0.99.48), *rcompanion* (ver. 2.4.21), and *stats* (ver. 4.2.2). The Shapiro‒Wilk test was used to assess the normality of the data distribution, whereas Bartlett’s test was used to evaluate variance homogeneity. The effects of BSFL fat administration (as a reference group) to broiler diets in comparison with those of selected plant or animal dietary fats were assessed through one-way ANOVA and Dunnets’ post hoc test for comparing several treatments with a reference group (BSFL). For nonnormally distributed data, Kruskal‒Wallis test, followed by the Dunn’s test was used with Benjamini‒Hochberg adjustment. Differences were considered significant at *P* < 0.05.

## Electronic supplementary material

Below is the link to the electronic supplementary material.

Supplementary Material 1

## Data Availability

All the raw data obtained in this study are deposited in an official repository (10.18150/IOUQUS).
