# Supplementary material for: Insect fat influences broiler performance, meat quality, and the cecal microbiota similarly to plant oils rather than animal fats
Source: Sci Rep. 2025 May 24;15:18086. doi: 10.1038/s41598-025-02820-3 (PMC12103505; doi:10.1038/s41598-025-02820-3)
Supplement: Supplementary file 1 — Supplementary Material 1 [file 41598_2025_2820_MOESM1_ESM.doc]

**Insect fat affects broiler performance, meat quality, and cecal microbiota similarly to plant oils rather than animal fats**

Muhammad Rumman Aslam^1^, Bartosz Kierończyk^1,*^, Mateusz Rawski^2^, Piotr Szymkowiak^1^, Kinga Stuper-Szablewska^3^, Paweł Kołodziejski^4^, Robert Mikuła^1^, Agata Dankowiakowska^5^, Damian Józefiak^1,*^

^1^Department of Animal Nutrition, Faculty of Veterinary Medicine and Animal Science, Poznań University of Life Sciences, Poznań, Poland

^2^Department of Zoology, Laboratory of Inland Fisheries and Aquaculture, Faculty of Veterinary Medicine and Animal Science, Poznań University of Life Sciences, Poznań, Poland

^3^Department of Chemistry, Faculty of Wood Technology, Poznań University of Life Sciences, Poznań, Poland

^4^Department of Animal Physiology, Biochemistry, and Biostructure, Poznań University of Life Sciences, Wołynska 33, 60-637 Poznań, Poland

^5^Department of Animal Physiology and Physiotherapy, Faculty of Animal Breeding and Biology, Bydgoszcz University of Science and Technology, Mazowiecka 28, 85-084 Bydgoszcz, Poland

***Corresponding authors:** Bartosz Kierończyk, email: [bartosz.kieronczyk@up.poznan.pl](mailto:bartosz.kieronczyk@up.poznan.pl), +48 61 848-72-22 and Damian Józefiak, email: [damian.jozefiak@up.poznan.pl](mailto:damian.jozefiak@up.poznan.pl), +48 61 848-72-27

**Supplementary Table 1**. Effect of the application of various dietary fat sources to broiler chicken diets on the total tract amino acid digestibility coefficient, % (d35).

|  | Treatment | | | | | | | | ^9^SEM | *p value* |
| --- | --- | --- | --- | --- | --- | --- | --- | --- | --- | --- |
|  | ^1^BSFL | ^2^SO | ^3^RO | ^4^PO | ^5^PKFD | ^6^PF | ^7^PL | ^8^BT |  |  |
| Histidine | 84.18 | 83.91 | 83.71 | 84.63 | 87.25 | 85.40 | 83.93 | 86.27 | 0.48 | *0.206* |
| Serine | 80.72 | 80.15 | 80.44 | 81.33 | 84.46 | 82.21 | 80.64 | 82.50 | 0.53 | *0.238* |
| Arginine | 88.94 | 88.98 | 88.93 | 89.92 | 91.36 | 90.63 | 88.72 | 90.39 | 0.34 | *0.222* |
| Glycine | 78.90 | 77.75 | 78.86 | 79.69 | 81.93 | 80.65 | 78.60 | 81.09 | 0.55 | *0.180* |
| Aspartic acid | 80.98 | 81.27 | 79.71 | 79.66 | 82.98 | 82.61 | 80.23 | 81.30 | 0.51 | *0.599* |
| Glutamic acid | 86.70 | 87.09 | 85.63 | 86.50 | 89.21 | 87.93 | 86.31 | 87.51 | 0.43 | *0.484* |
| Threonine | 79.88 | 79.00 | 79.53 | 79.55 | 83.09 | 81.01 | 79.38 | 81.85 | 0.54 | *0.231* |
| Alanine | 83.07 | 82.83 | 81.30 | 82.13 | 86.37 | 83.81 | 82.85 | 84.32 | 0.57 | *0.332* |
| Proline | 83.09 | 82.27 | 81.29 | 82.61 | 85.06 | 83.48 | 82.03 | 84.12 | 0.51 | *0.432* |
| Lysine | 86.33 | 87.21 | 86.10 | 85.66 | 88.64 | 87.98 | 86.71 | 87.53 | 0.41 | *0.599* |
| Tyrosine | 83.11 | 82.23 | 82.61 | 84.89 | 87.11 | 84.89 | 83.04 | 85.32 | 0.56 | *0.165* |
| Valine | 82.09 | 81.51 | 80.80 | 81.96 | 84.73 | 83.16 | 82.13 | 84.11 | 0.55 | *0.182* |
| Isoleucine | 83.38 | 83.02 | 82.29 | 83.43 | 86.30 | 84.80 | 83.23 | 85.01 | 0.53 | *0.242* |
| Leucine | 84.85 | 84.45 | 83.14 | 84.50 | 87.65 | 85.65 | 84.32 | 86.16 | 0.53 | *0.324* |
| Phenylalanine | 85.44 | 84.79 | 84.81 | 86.53 | 88.72 | 86.91 | 84.93 | 87.06 | 0.48 | *0.252* |

^1^BSFL – basal diet with 100% black soldier fly (*Hermetia illucens*) larval fat; ^2^SO – basal diet with 100% soybean oil; ^3^RO – basal diet with 100% rapeseed oil; ^4^PO – basal diet with 100% palm oil; ^5^PKFD – basal diet with 100% palm kernel fat distillers; ^6^PF – basal diet with 100% poultry fat; ^7^PL – basal diet with 100% pig lard; ^8^BT – basal diet with 100% beef tallow; ^9^SEM – standard error of the mean; ^10^BWG – body weight gain; ^11^FI – feed intake; ^12^FCR – feed conversion ratio.

The means represent the 10 birds’ excreta/digesta pooled by 2 (*n* = 5).

Analysis of variance (ANOVA) or Kruskal-Wallis test depending on the normal distribution of data provides the probability level (*P value*) exhibited in the last column.

**Supplementary Table 2.** Effects of various dietary fat applications in broiler chicken diets on the selected internal organ and gastrointestinal tract segment weights (% of BW) and length (cm/kg BW).

|  | Treatment | | | | | | | | ^9^SEM | *P value* |
| --- | --- | --- | --- | --- | --- | --- | --- | --- | --- | --- |
|  | ^1^BSFL | ^2^SO | ^3^RO | ^4^PO | ^5^PKFD | ^6^PF | ^7^PL | ^8^BT |  |  |
| Selected organ weights, % of BW | | | | | | | | | | |
| Duodenum | 0.79 | 0.81 | 0.86 | 0.91 | 0.77 | 0.80 | 0.87 | 0.86 | 0.01 | *0.098* |
| Jejunum | 1.47 | 1.49 | 1.59 | 1.47 | 1.20 ^*^ | 1.46 | 1.50 | 1.55 | 0.03 | *<0.001* |
| Ileum | 1.13 | 1.16 | 1.21 | 1.09 | 0.95 | 1.05 | 1.15 | 1.17 | 0.02 | *0.691* |
| Cecum | 0.28 | 0.34 | 0.29 | 0.31 | 0.37 | 0.30 | 0.30 | 0.28 | 0.01 | *0.613* |
| Proventriculus | 0.33 | 0.31 | 0.37 | 0.35 | 0.30 | 0.30 | 0.32 | 0.31 | 0.01 | *0.055* |
| Gizzard | 0.96 | 1.01 | 0.92 | 0.93 | 1.03 | 0.88 | 0.95 | 0.89 | 0.02 | *0.252* |
| Heart | 0.48 | 0.54 | 0.51 | 0.53 | 0.54 | 0.51 | 0.51 | 0.52 | 0.01 | *0.397* |
| Pancreas | 0.25 | 0.24 | 0.26 | 0.23 | 0.26 | 0.25 | 0.27 | 0.27 | 0.01 | *0.385* |
| Liver | 2.33 | 2.49 | 2.65 | 2.55 | 2.56 | 2.57 | 2.58 | 2.63 | 0.04 | *0.262* |
| Immune organ index, % of BW | | | | | | | | | | |
| Spleen | 0.09 | 0.11 | 0.09 | 0.11 | 0.13 | 0.10 | 0.11 | 0.10 | <0.01 | *0.186* |
| Bursa of Fabricius | 0.14 | 0.10 | 0.10 | 0.12 | 0.14 | 0.11 | 0.11 | 0.12 | 0.01 | *0.166* |
| Thymus | 0.24 | 0.24 | 0.25 | 0.24 | 0.27 | 0.29 | 0.27 | 0.22 | 0.01 | *0.067* |
| Selected GIT segment lengths, cm ⋅ kg^-1^ BW | | | | | | | | | | |
| Duodenum | 17.70 | 17.45 | 17.28 | 17.51 | 16.25 | 15.44 | 16.70 | 16.51 | 0.25 | *0.259* |
| Jejunum | 41.18 | 40.55 | 39.10 | 41.99 | 38.09 | 36.92 | 40.53 | 39.17 | 0.55 | *0.222* |
| Ileum | 41.70 | 41.88 | 39.51 | 42.51 | 37.26 | 36.51 | 41.81 | 39.68 | 0.63 | *0.080* |
| Cecum | 8.20 | 8.23 | 7.37 | 7.78 | 8.43 | 7.27 | 7.60 | 7.44 | 0.13 | *0.089* |

^*^ p < 0.05, ^**^ p < 0.01, ^***^ p < 0.001 - the reference group (BSFL) is significantly different within a row when asterisks are present.

^1^BSFL – basal diet with 100% black soldier fly (*Hermetia illucens*) larval fat; ^2^SO – basal diet with 100% soybean oil; ^3^RO – basal diet with 100% rapeseed oil; ^4^PO – basal diet with 100% palm oil; ^5^PKFD – basal diet with 100% palm kernel fat distillers; ^6^PF – basal diet with 100% poultry fat; ^7^PL – basal diet with 100% pig lard; ^8^BT – basal diet with 100% beef tallow; ^9^SEM – standard error of the mean; Means represent 1 bird randomly chosen from each pen (*n* = 10).

Analysis of variance (ANOVA) or Kruskal-Wallis test depending on the normal distribution of data provides the probability level (*P value*) exhibited in the last column.

**Supplementary Table 3.** Effects of the dietary fat application of various broiler chicken diets on the histomorphology changes in the liver.

|  | Treatment | | | | | | | | ^9^SEM | *P value* |
| --- | --- | --- | --- | --- | --- | --- | --- | --- | --- | --- |
|  | ^1^BSFL | ^2^SO | ^3^RO | ^4^PO | ^5^PKFD | ^6^PF | ^7^PL | ^8^BT |  |  |
| Parenchymal eclipse | 0.90 | 1.90 | 0.70 | 1.30 | 1.00 | 1.30 | 0.50 | 0.40 | 0.13 | 0.201 |
| Presence of vacuoles | 0.10 | 0.00 | 0.00 | 0.20 | 0.00 | 0.00 | 0.00 | 0.00 | 0.02 | 0.157 |
| Number of congestions | 1.60 | 0.80 | 1.30 | 1.00 | 1.20 | 0.70 | 0.80 | 0.50 | 0.12 | 0.506 |
| Number of necrosis | 0.40 | 0.38 | 0.44 | 0.10 | 0.00 | 0.00 | 0.00 | 0.00 | 0.05 | 0.012 |
| Number of fibrosis | 1.70 | 1.20 | 0.10 | 0.50 | 0.80 | 1.40 | 0.70 | 0.80 | 0.15 | 0.146 |

Assessment was performed with the use of a 5-point (0–4) scale, where 0 represents no changes, 1 represents slight histopathology present in less than 25% of fields, 2 represents mild histopathology present in less than 50% of fields, 3 represents moderate histopathology present in less than 75% of fields, and 4 represents severe histopathology present in more than 75% of fields.

^1^BSFL – basal diet with 100% black soldier fly (*Hermetia illucens*) larval fat; ^2^SO – basal diet with 100% soybean oil; ^3^RO – basal diet with 100% rapeseed oil; ^4^PO – basal diet with 100% palm oil; ^5^PKFD – basal diet with 100% palm kernel fat distillers; ^6^PF – basal diet with 100% poultry fat; ^7^PL – basal diet with 100% pig lard; ^8^BT – basal diet with 100% beef tallow; ^9^SEM – standard error of the mean; Means represent 1 bird randomly chosen from each pen (*n* = 10).

Analysis of variance (ANOVA) or Kruskal-Wallis test depending on the normal distribution of data provides the probability level (*P value*) exhibited in the last column.

ND = not detected

**Supplementary Table 4**.Effects of the application of various dietary fat sources to broiler chicken diets on the selected microbial populations (log CFU/g digesta) in the cecal digesta determined by DAPI staining and fluorescent in situ hybridization (FISH).

| Item | Treatment | | | | | | | | ^9^SEM | | *P value* | |
| --- | --- | --- | --- | --- | --- | --- | --- | --- | --- | --- | --- | --- |
|  | ^1^BSFL | ^2^SO | ^3^RO | ^4^PO | ^5^PKFD | ^6^PF | ^7^PL | ^8^BT | |  | |  |
| ^10^DAPI | 10.6 | 10.7 | 10.7 | 10.4 | 10.7 | 10.8 ^*^ | 10.8 ^*^ | 10.7 | | 0.04 | | *0.047* |
| *Bacteroides*-*Prevotella* cluster | 9.28 | 7.63 | 9.10 | 6.93 | 7.36 | 8.79 | 8.84 | 8.56 | | 0.29 | | *0.073* |
| *Clostridium leptum* subgroup | 7.80 | 8.51 | 9.09 | 7.75 | 8.78 | 9.31 | 9.52 | 8.78 | | 0.19 | | *0.084* |
| *Clostridium perfringens* | 7.42 | 7.32 | 7.22 | 7.20 | 6.89 | 7.76 | 6.61 | 5.50 | | 0.35 | | *0.652* |
| Enterobacteriaceae | 8.56 | 6.18 | 7.25 | 6.52 | 8.29 | 8.36 | 9.13 | 7.70 | | 0.23 | | *0.101* |
| *Clostridium coccoides*-*Eubacterium rectale* cluster | 9.76 | 9.71 | 9.65 | 9.63 | 9.70 | 8.51 | 9.83 | 9.72 | | 0.13 | | *0.864* |
| *Lactobacillus* sp./*Enterococcus* sp. | 7.70 | 8.36 | 8.62 | 7.58 | 6.24 | 9.13 | 9.12 | 8.49 | | 0.27 | | *0.265* |

^*^ p < 0.05, ^**^ p < 0.01, ^***^ p < 0.001 - the reference group (BSFL) is significantly different within a row when asterisks are present.

^1^BSFL – basal diet with 100% black soldier fly (*Hermetia illucens*) larval fat; ^2^SO – basal diet with 100% soybean oil; ^3^RO – basal diet with 100% rapeseed oil; ^4^PO – basal diet with 100% palm oil; ^5^PKFD – basal diet with 100% palm kernel fat distillers; ^6^PF – basal diet with 100% poultry fat; ^7^PL – basal diet with 100% pig lard; ^8^BT – basal diet with 100% beef tallow; ^9^SEM – standard error of the mean; ^10^DAPI – total number of bacteria determined by 4',6-diamidino-2-phenylindole staining; Means represent 1 bird randomly chosen from each pen pooled by two (*n* = 5).

Analysis of variance (ANOVA) or Kruskal-Wallis test depending on the normal distribution of data provides the probability level (*P value*) exhibited in the last column.

**Supplementary Table 5.** Effects of various dietary fat applications in broiler chicken diets on the selected carcass traits.

|  | Treatment | | | | | | | | ^9^SEM | *P value* |
| --- | --- | --- | --- | --- | --- | --- | --- | --- | --- | --- |
|  | ^1^BSFL | ^2^SO | ^3^RO | ^4^PO | ^5^PKFD | ^6^PF | ^7^PL | ^8^BT |  |  |
| Final body weight, g | 2501 | 2487 | 2502 | 2518 | 2516 | 2536 | 2492 | 2516 | 16.39 | *0.700* |
| Carcass weight, g | 1902 | 1831 | 1877 | 1887 | 1935 | 1899 | 1870 | 1907 | 13.06 | *0.472* |
| Carcass yield, % | 76.05 | 73.61 | 75.02 | 74.96 | 76.92 | 74.86 | 75.06 | 75.77 | 0.18 | *0.305* |
| Breast yield, % | 29.3 | 28.3 | 30.1 | 30.0 | 29.0 | 28.0 | 28.5 | 30.9 | 0.43 | *0.015* |
| Leg quarters yield, % | 23.84 | 24.67 | 24.88 | 24.76 | 24.63 | 23.84 | 24.34 | 23.81 | 0.11 | *0.216* |
| Drumstick yield, % | 11.90 | 12.2 | 11.29 | 11.73 | 11.69 | 11.53 | 11.51 | 11.07 ^*^ | 0.07 | *0.004* |
| Thigh’s yield, % | 11.93 | 12.44 | 13.59 ^*^ | 13.03 ^*^ | 12.95 | 12.31 | 12.83 | 12.74 | 0.10 | *0.010* |
| Wings yield, % | 9.79 | 9.84 | 8.98 | 9.68 | 9.56 | 9.39 | 9.36 | 9.27 | 0.08 | *0.159* |
| Giblet’s yield, % | 9.30 | 10.86 | 11.10 | 10.41 | 10.32 | 10.79 | 10.67 | 10.78 | 0.13 | *0.073* |

^*^ p < 0.05, ^**^ p < 0.01, ^***^ p < 0.001 - the reference group (BSFL) is significantly different within a row when asterisks are present.

^1^BSFL – basal diet with 100% black soldier fly (*Hermetia illucens*) larval fat; ^2^SO – basal diet with 100% soybean oil; ^3^RO – basal diet with 100% rapeseed oil; ^4^PO – basal diet with 100% palm oil; ^5^PKFD – basal diet with 100% palm kernel fat distillers; ^6^PF – basal diet with 100% poultry fat; ^7^PL – basal diet with 100% pig lard; ^8^BT – basal diet with 100% beef tallow; ^9^SEM – standard error of the mean; Means represent 1 bird randomly chosen from each pen (*n* = 10).

Analysis of variance (ANOVA) or Kruskal-Wallis test depending on the normal distribution of data provides the probability level (*P value*) exhibited in the last column.

Giblets = heart + gizzard + liver

**Supplementary Table 6.** Effects of various dietary fat applications in broiler chicken diets on the fatty acid profile of the liver (g/100 g FA).

| Component | Treatments | | | | | | | | ^9^SEM | *p value* |
| --- | --- | --- | --- | --- | --- | --- | --- | --- | --- | --- |
|  | ^1^BSFL | ^2^SO | ^3^RO | ^4^PO | ^5^PKFD | ^6^PF | ^7^PL | ^8^BT |  |  |
| *Saturated fatty acids* | | | | | | | | | | |
| C14:0 | 0.45 | 0.46 | 0.46 | 0.46 | 0.44 | 0.46 | 0.46 | 0.46 | 0.003 | *0.786* |
| C16:0 | 28.8 | 27.7 ^***^ | 28.9 | 28.3 ^***^ | 28.8 | 28.1 ^***^ | 28.8 | 28.8 | 0.049 | *<0.001* |
| C18:0 | 9.24 | 9.35 | 9.29 | 9.34 | 9.36 | 9.31 | 9.67 | 9.31 | 0.028 | *0.452* |
| C20:0 | 0.08 | 0.11 | 0.09 | 0.11 | 0.08 | 0.09 | 0.10 | 0.08 | 0.009 | *0.963* |
| C22:0 | 0.09 | 0.09 | 0.09 | 0.09 | 0.09 | 0.08 | 0.09 | 0.09 | 0.001 | *0.772* |
| *Unsaturated fatty acids* | | | | | | | | | | |
| C14:1 | 0.06 | 0.06 | 0.06 | 0.06 | 0.07 | 0.06 | 0.05 | 0.06 | 0.001 | *0.105* |
| C16:1 n7 | 2.47 | 2.48 | 2.50 | 2.48 | 2.08 ^***^ | 2.10 ^***^ | 2.10 ^***^ | 2.44 | 0.029 | *<0.001* |
| C18:1 n9 | 46.8 | 47.5 | 46.7 | 47.2 | 46.5 | 47.6 | 46.8 | 46.8 | 0.049 | *0.423* |
| C18:2 n6 | 9.11 | 9.12 | 9.12 | 9.10 | 9.09 | 9.08 | 9.09 | 9.11 | 0.007 | *0.816* |
| C18:3 n3 | 0.00 | 0.22 ^***^ | 0.00 | 0.00 | 0.49 ^***^ | 0.34 ^***^ | 0.05 | 0.00 | 0.022 | *<0.001* |
| C18:3 n6 | 0.33 | 0.33 | 0.32 | 0.33 | 0.32 | 0.32 | 0.32 | 0.32 | 0.002 | *0.959* |
| C20:2 | 2.24 | 2.22 | 2.18 | 2.20 | 2.21 | 2.17 | 2.19 | 2.22 | 0.014 | *0.948* |
| C20:4 | 0.35 | 0.35 | 0.33 | 0.35 | 0.36 | 0.35 | 0.34 | 0.34 | 0.003 | *0.516* |
| *Summarized fatty acids* | | | | | | | | | | |
| SFA | 38.6 | 37.7 ^***^ | 38.8 | 38.3 | 38.9 | 38.0 ^***^ | 39.1 ^*^ | 38.7 | 0.057 | *<0.001* |
| UFA | 61.4 | 62.3 ^***^ | 61.2 | 61.7 | 61.1 | 62.0 ^***^ | 60.9 ^*^ | 61.3 | 0.057 | *<0.001* |
| MUFA | 49.3 | 50.0 ^***^ | 49.3 | 49.7 | 48.7 ^***^ | 49.7 | 48.9 | 49.3 | 0.060 | *<0.001* |
| PUFA | 12.0 | 12.2 ^*^ | 12.0 | 12.0 | 12.5 ^***^ | 12.3 ^*^ | 12.0 | 12.0 | 0.028 | *<0.001* |
| PUFA/SFA | 0.31 | 0.32 | 0.31 | 0.31 | 0.32 | 0.32 | 0.31 | 0.31 | 0.001 | *0.213* |
| n6 | 9.43 | 9.45 | 9.44 | 9.43 | 9.42 | 9.41 | 9.42 | 9.43 | 0.007 | *0.854* |
| n3 | 0.00 | 0.22 ^***^ | 0.00 | 0.00 | 0.49 ^***^ | 0.34 ^***^ | 0.05 | 0.00 | 0.022 | *<0.001* |
| AI | 0.52 | 0.50 ^***^ | 0.52 | 0.51 ^***^ | 0.52 | 0.50 ^***^ | 0.52 | 0.52 | 0.001 | *<0.001* |
| TI | 1.31 | 1.23 ^***^ | 1.32 | 1.29 | 1.27 ^***^ | 1.24 ^***^ | 1.33 | 1.31 | 0.004 | *<0.001* |

^*^ p < 0.05, ^**^ p < 0.01, ^***^ p < 0.001 - the reference group (BSFL) is significantly different within a row when asterisks are present.

^1^BSFL – basal diet with 100% black soldier fly (*H. illucens*) larval fat; ^2^SO – basal diet with 100% soybean oil; ^3^RO – basal diet with 100% rapeseed oil; ^4^PO – basal diet with 100% palm oil; ^5^PKFD – basal diet with 100% palm kernel fat distillate; ^6^PF – basal diet with 100% poultry fat; ^7^PL – basal diet with 100% pig lard; ^8^BT – basal diet with 100% beef tallow; ^9^SEM – standard error of the mean; Means represent 1 bird randomly chosen from each pen (*n* = 10). Analysis of variance (ANOVA) or the Kruskal‒Wallis test, depending on the normal distribution of the data, provides the probability level (*P value*) presented in the last column.

**Supplementary Table 7.** Effects of the application of various dietary fat sources to broiler chicken diets on the selected sensory attributes of chicken breast filets.

| Item | Treatments | | | | | | | | SEM | *P value* |
| --- | --- | --- | --- | --- | --- | --- | --- | --- | --- | --- |
|  | ^1^BSFL | ^2^SO | ^3^RO | ^4^PO | ^5^PKFD | ^6^PF | ^7^PL | ^8^BT |  |  |
| Visual sense | | | | | | | | | | |
| Color intensity | 2.8 | 1.8 | 2.6 | 2.3 | 2.9 | 3.0 | 2.6 | 2.4 | 0.11 | *0.128* |
| Smell sense | | | | | | | | | | |
| Overall odor | 3.1 | 2.4 | 3.0 | 2.8 | 3.1 | 2.8 | 2.9 | 3.1 | 0.11 | *0.742* |
| Animal/Barn odor | 2.4 | 2.3 | 2.6 | 2.3 | 2.5 | 2.3 | 2.1 | 2.4 | 0.14 | *0.997* |
| Metallic odor | 1.1 | 1.2 | 1.2 | 1.0 | 1.1 | 1.0 | 1.1 | 1.3 | 0.04 | *0.453* |
| Cooked chicken odor | 4.2 | 3.3 | 3.3 | 3.2 | 3.1 | 2.9 ^*^ | 3.2 | 2.9 ^*^ | 0.11 | *0.008* |
| Texture sense | | | | | | | | | | |
| Juiciness | 2.7 | 3.3 | 3.2 | 3.1 | 3.4 | 3.1 | 2.5 | 3.1 | 0.13 | *0.751* |
| Hardness | 2.3 | 2.9 | 2.3 | 2.4 | 2.3 | 2.1 | 1.7 | 2.4 | 0.10 | *0.211* |
| Adhesiveness | 1.6 | 1.5 | 1.4 | 2.1 | 1.7 | 2.3 | 1.9 | 2.1 | 0.09 | *0.100* |
| Taste sense | | | | | | | | | | |
| Bitter taste | 1.1 | 1.4 | 1.4 | 1.4 | 1.6 | 1.5 | 1.7 | 1.4 | 0.08 | *0.820* |
| Sour taste | 1.4 | 1.3 | 1.4 | 1.5 | 1.5 | 1.4 | 1.2 | 1.6 | 0.07 | *0.930* |
| Sweet taste | 2.4 | 1.5 | 1.6 | 1.4 | 1.4 | 1.7 | 1.8 | 1.4 | 0.10 | *0.065* |
| Metallic flavor | 1.2 | 1.4 | 1.6 | 1.5 | 1.8 | 1.8 | 1.6 | 1.7 | 0.09 | *0.690* |
| Chicken flavor | 4.2 | 3.6 | 3.4 | 3.5 | 3.4 | 3.6 | 3.6 | 3.0 | 0.11 | *0.052* |
| Aftertaste | 3.4 | 3.0 | 2.9 | 3.3 | 3.4 | 3.5 | 3.6 | 3.0 | 0.10 | *0.435* |

^*^ p < 0.05, ^**^ p < 0.01, ^***^ p < 0.001 - the reference group (BSFL) is significantly different within a row when asterisks are present.

^1^BSFL – basal diet with 100% black soldier fly (*Hermetia illucens*) larval fat; ^2^SO – basal diet with 100% soybean oil; ^3^RO – basal diet with 100% rapeseed oil; ^4^PO – basal diet with 100% palm oil; ^5^PKFD – basal diet with 100% palm kernel fat distillers; ^6^PF – basal diet with 100% poultry fat; ^7^PLbasal diet with 100% pig lard; ^8^BT – basal diet with 100% beef tallow; ^9^SEM – standard error of the mean; Means represent 1 bird randomly chosen from each pen (*n* = 10).

Analysis of variance (ANOVA) or Kruskal-Wallis test depending on the normal distribution of data provides the probability level (*P value*) exhibited in the last column.

Assessment with use of a 5-point (1-5) scale, where in terms of visual sense 0 represents pale color and 5 dark meat; in the case of smell sense 1 represents undetected odor, and 5 – very intensive odor; texture sense was determined as fallow, juiciness (1 – very juicy, 5 – a lack of juiciness), hardness (1 – very tender, 5 – very hard), adhesiveness (1 – a lack of adhesiveness, 5 – peanut butter); in the case of taste sense 1 represents undetected taste, and 5 – intensive taste.

**Supplementary Table 8.** Selected oligonucleotide probes used in the study.

| Probe | Target | Sequence (5’-3’) | Reference |
| --- | --- | --- | --- |
| Bacto303 | *Bacteroides, Prevotella* | CCA ATG TGG GGG ACC TT | Manz et al.^1^ |
| Cperf191 | *Clostridium perfringens* | GTA GTA AGT TGG TTT CCT CG | Fallani et al.^2^ |
| Enter1432 | Enterobacteriaceae | CTT TTG CAA CCC ACT | Sghir et al.^3^ |
| Erec482 | *Clostridium coccoides – Eubacterium rectale* cluster | GCT TCT TAG TCA RGT ACC G | Sghir et al.^3^, Welling et al.^4^ |
| Clept1240 | *Clostridium leptum* | GTT TTR TCA ACG GCA GTC | Sghir et al.^3^ |
| Lab158 | *Lactobacillus* sp./*Enterococcus* sp. | GGT ATT AGC AYC TGT TTC CA | Harmsen et al.^5^ |

^1^Manz, W.; Szewczyk, U.; Ericsson, P.; Amann, R.; Schleifer, K. & Stenström, T. *In situ* identiﬁcation of bacteria in drinking water and adjoining bioﬁlms by hybridization with 16S and 23S rRNA-directed ﬂuorescent oligonucleotide probes. *Appl. Environ. Microbiol.* **59**, 2293–2298 (1993).

^2^Fallani, M.; Rigottier-Gois, L.; Aguilera, M.; Bridonneau, C.; Collignon, A.; Edwards, C.A.; Corthier, G. & Doré, J. *Clostridium diﬃcile* and *Clostridium perfringens* species detected in infant faecal microbiota using 16SrRNA targeted probes. *J. Microbiol. Methods* **67**, 150–161 (2006).

^3^Sghir, A.; Gramet, G.; Suau, A.; Rochet, V.; Pochart, P. & Dore, J. Quantification of bacterial groups within human fecal flora by oligonucleotide probe hybridization. *Appl. Environ. Microbiol*. **66**, 2263–2266 (2000).

^4^Welling, G.W.; Wildeboer-Veloo, L.; Raangs, G.C.; Franks, A.H.; Jansen, G.J.; Tonk, R.H.; Degener, J.E. & Harmsen, H.J. Variations of bacterial populations in human faeces measured by FISH with group-speciﬁc16S rRNA-targeted oligonucleotide probes. *Biosci. Microﬂora*, **19**, 79–84 (2000).

^5^Harmsen, H.J.; Elﬀerich, P.; Schut, F. & Welling, G.W. A 16S rRNA-targeted probe for detection of lactobacilli and enterococci in faecal samples by ﬂuorescent in situ hybridization. *Microb. Ecol. Health Dis.* **11**, 3–12 (1999).
